# Structural Investigation of Park’s Nucleotide on Bacterial Translocase MraY: Discovery of Unexpected MraY Inhibitors

**DOI:** 10.1038/srep31579

**Published:** 2016-08-17

**Authors:** Kuo-Ting Chen, Po-Ting Chen, Cheng-Kun Lin, Lin-Ya Huang, Chia-Ming Hu, Yi-Fan Chang, Hua-Ting Hsu, Ting-Jen R. Cheng, Ying-Ta Wu, Wei-Chieh Cheng

**Affiliations:** 1 Genomics Research Center, Academia Sinica, No. 128 Academia Road, Section 2, Nankang District, Taipei, 11529, Taiwan

## Abstract

Systematic structural modifications of the muramic acid, peptide, and nucleotide moieties of Park’s nucleotide were performed to investigate the substrate specificity of *B. subtilis* MraY (MraY_BS_). It was found that the simplest analogue of Park’s nucleotide only bearing the first two amino acids, l-alanine-*iso*-d-glutamic acid, could function as a MraY_BS_ substrate. Also, the acid group attached to the *C*α of *iso*-d-glutamic acid was found to play an important role for substrate activity. Epimerization of the *C*4-hydroxyl group of muramic acid and modification at the 5-position of the uracil in Park’s nucleotide were both found to dramatically impair their substrate activity. Unexpectedly, structural modifications on the uracil moiety changed the parent molecule from a substrate to an inhibitor, blocking the MraY_BS_ translocation. One unoptimized inhibitor was found to have a *K*_i_ value of 4 ± 1 μM against MraY_BS_, more potent than tunicamycins.

Peptidoglycan is a polymer consisting of sugars and amino acids that forms the bacterial cell wall. Interrupting the biosynthesis of peptidoglycan can devastate bacterial growth and survival due to the critical role it plays in maintaining cell shape and protecting bacteria from internal osmotic pressure^1^,^2^. One of the enzymes involved in bacterial cell wall biosynthesis, MraY is an integral membrane protein that catalyzes the transfer of the monophospho-MurNAc-pentapeptide moiety from Park’s nucleotide (UDP-MurNAc-pentapeptide) onto the undecaprenyl phosphate, to give Lipid I with concomitant release of UMP (Fig. 1). MraY is an attractive antibacterial target being essential for bacterial growth; highly conserved across many bacterial species; and without a eukaryotic counterpart^3^,^4^,^5^,^6^.

One major class of MraY inhibitors, known as nucleoside antibiotics, shares a uridine nucleoside as a common moiety with Park’s nucleotide^6^,^7^,^8^,^9^,^10^. Accordingly, an understanding of the interactions between Park’s nucleotide and MraY might be useful for the design of new MraY inhibitors. Recent disclosure of an apo crystal structure of MraY from *Aquifex aeolicus* (MraY_AA_) shows the overall architecture of this interesting enzyme^11^. However, due to the lack of available complex crystal structure, detailed mechanisms or interactions between substrates or inhibitors toward MraY remain to be explored. Although some brief substrate studies of Park’s nucleotide toward MraY have been reported, their scope is limited to the structural diversity accessible by biocatalysis^12^. Obviously, the substrate study of MraY is hampered by difficulties to acquire the structurally complex substrates. Chemical synthesis seems to be the most straightforward approach towards the generation of pure and systematically modified samples of various desired molecules for testing against MraY.

To more thoroughly investigate how structural modification of Park’s nucleotide affects MraY substrate recognition, we first sought to identify a proper polyprenyl phosphate substrate that would be conserved for all the Park’s nucleotide analogues tested. In our preliminary HPLC-based MraY activity study, NBD-Park’s nucleotide **6** was completely consumed in 1 h when undecaprenyl phosphate (C55P) was applied as a polyprenyl phosphate substrate in our hands (Supplementary Figure 1)^13^. In contrast, other polyprenyl phosphates with a shorter length or different configurations still can be recognized as a MraY substrate but their substrate activity is much weaker than undecaprenyl phosphate (C55P) (Supplementary Table 1). Our observation of this broad substrate specificity of MraY is consistent with previous studies in the combined MraY-MurG system or membrane fractions containing both MraY and MurG^14^,^15^,^16^. According to our results, C55P was chosen as the substrate coupling partner for all the Park’s nucleotide analogues studies, and the substrate activity was measured after 1 h reaction for convenient purposes. Moreover, it was decided not to modify the pyrophosphate group as it is at this position that translocation occurs.

Herein, we describe the systematic preparation of Park’s nucleotides with varying three parts including the peptide, *N*-substituted muramic acid, and uridine moieties for evaluation as MraY_BS_ substrates (Fig. 2). This information will provide us with the essential moieties and the specificity requirements of the MraY for Park’s nucleotide analogues, as an effort toward development of new inhibitors.

## Results and Discussions

### Preparation of Park’s nucleotide analogues and evaluation of their substrate activity

**Figure 3:**
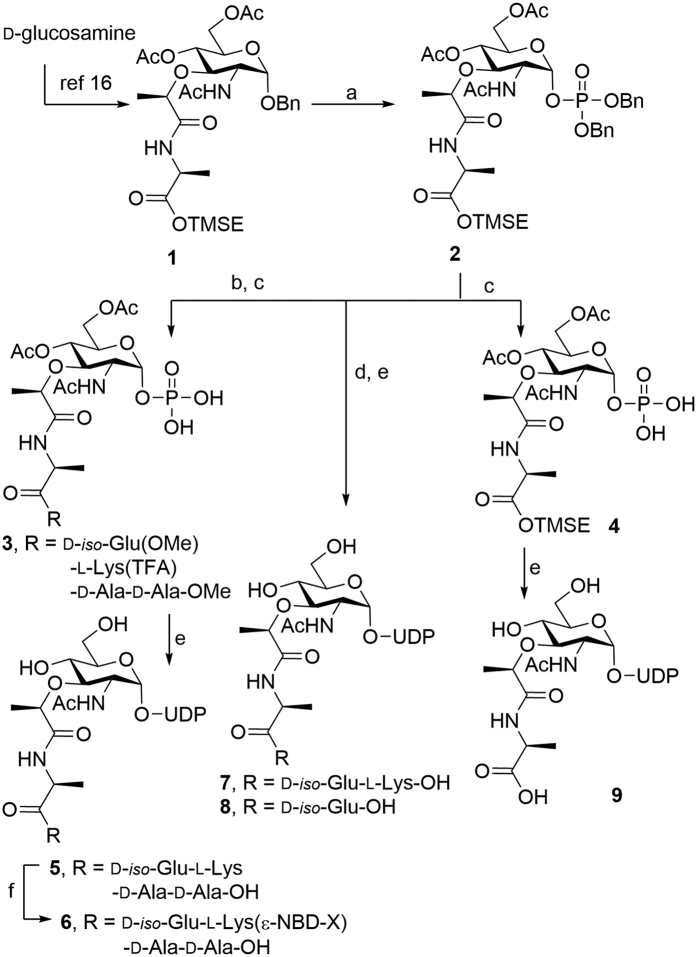
Synthesis of Park’s nucleotide analogues (**5**–**9**). Reagents and conditions: (**a**) i. Pd(OH)_2_/H_2_, THF, RT, 24 h, ii. *i*Pr_2_NP(OBn)_2_, 1*H*-tetrazole, CH_2_Cl_2_, ACN, 0 °C, 2 h, iii. *t*BuOOH, −40 °C to RT, 1 h, 71% over three steps; (**b**) i. TBAF, THF, RT, 2 h, ii. H-d-*iso*-Glu(OMe)-l-Lys(TFA)-d-Ala-d-Ala-OMe, PyBOP, DIEA, THF, CH_2_Cl_2_, RT, 0.5 h, 85%; (**c**) Pd(OH)_2_/H_2_, MeOH, RT, 1 h, 79% (for **3**) and 75% (for **4**); (**d**) i. TBAF, THF, RT, 2 h, ii. H-d-*iso*-Glu(OMe)-l-Lys(TFA)-OMe (for **7**); H-d-*iso*-Glu(OMe)-OMe (for **8**), PyBOP, DIEA, THF, CH_2_Cl_2_, RT, 0.5 h, iii. Pd(OH)_2_/H_2_, MeOH, RT, 1 h; (**e**) i. UMP-morpholine-*N,N′*-dicyclohexylcarboxamidine salt, 1*H*-tetrazole, pyridine, 4 Å molecular sieves, 0 °C to RT, 24 h, ii. LiOH, MeOH, RT, 4 h, 35% (for **5**), 69% (for **9**) over two steps, and 46% (for **7**), 43% (for **8**) over five steps; (**f** ) NBD-X-OSu, NaHCO_3_, H_2_O, DMF, RT, 2 h, 88%.

As shown in Fig. 3, *O-*debenzylation of **1** followed by a phosphorylation and phosphitylation/oxidation sequence gave the phosphate **2** in 71% yield over three steps^17^. Compound **4** was obtained via the debenzylation of **2**. Finally, conjugation of **4** with activated UMP-morpholine-*N,N*’-dicyclohexyl carboxamidine salt and global deprotection under basic conditions gave Park’s nucleotide **9** in 69% yield. For the preparation of **5**, selective deprotection of the trimethylsilyl ethyl ester (TMSE) in **2** by treatment with TBAF in THF, followed by coupling with H-d-*iso*-Glu(OMe)-l-Lys-(TFA)-d-Ala-d-Ala(OMe) and debenzylation gave the corresponding **3** in 67% yield over three steps. Compound **3** was then coupled with activated UMP-morpholine-*N,N*’-dicyclohexylcarboxamidine salt, followed by global deprotection under basic conditions gave Park’s nucleotide **5** in 35% yield over two steps. A fluorescent probe **6** was prepared from **5** by conjugating a nitronbenzoxadiazole (NBD) fluorophore at the terminal amine site of lysine on the peptide stem in 88% yield. Compounds **7** and **8** were similarly prepared (Fig. 3).

**Figure 4:**
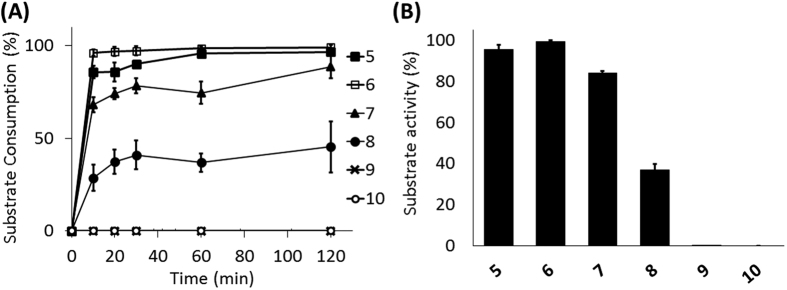
Evaluation of the substrate activity of Park’s nucleotide analogues 5–10 toward MraY_BS_. (**A**) The reactions were analysed in the HPLC-based MraY functional assay as described in Methods and the progresses were measured at 0, 10, 20, 30, 60 and 120 min. (**B**) The substrate activity of **5**–**10** was determined by the substrate consumption after 1 h reaction time. All experiments were repeated in triplicate (Supplementary Figure 3).

The substrate activity study of **5**–**10** toward MraY_BS_ was performed using the HPLC-based MraY functional assay. Substrate consumption curves of **5**–**10** were shown in Fig. 4A. Compounds **5**–**8** were recognized as a MraY_BS_ substrate, but **9** and **10** were not. The similar curves of **5** and **6** suggest that the NBD-fluorophore attaching to the side chain of Lys on the pentapeptide stem of Park’s nucleotide does not cause any significant effect on its substrate activity (Supplementary Figures 1 and 2). Compound **7**, lacking the terminal two amino acids (d-Ala-d-Ala), was only slightly less active than **5** (17% activity reduced after 1 h reaction, Fig. 4B), showing that the d-Ala-d-Ala moiety is not essential for MraY_BS_ recognition. The previous study reported by Hammes and Neuhaus pointed out that **7** is a much weaker substrate than **5** when intrinsic membrane fractions are used as a source of lipidphosphate and enzyme^12^. In our conditions, only the purified enzyme and two pure substrates were utilized, and Park’s nucleotide analogue was the limiting reagent compared to the other substrate C55P. Both individual studies show different degrees of the substrate activity loss that might be attributed to several factors such as enzyme activity, substrate ratio and assay platform. Compound **8**, similar to **5** but lacking the terminal three amino acids, was a weak substrate (40% activity remained after 1 h reaction, Fig. 4B). Moreover, **9** (bearing only one amino acid (l-Ala)) and UDP-GlcNAc (**10**) were not substrates under these assay conditions, showing that this 3-*O*-lactyl-tripeptide (d-Lac-l-Ala-γ-d-Gln-l-Lys) moiety in Park’s nucleotide is important for the MraY_BS_ catalyzing process.

**Figure 5:**
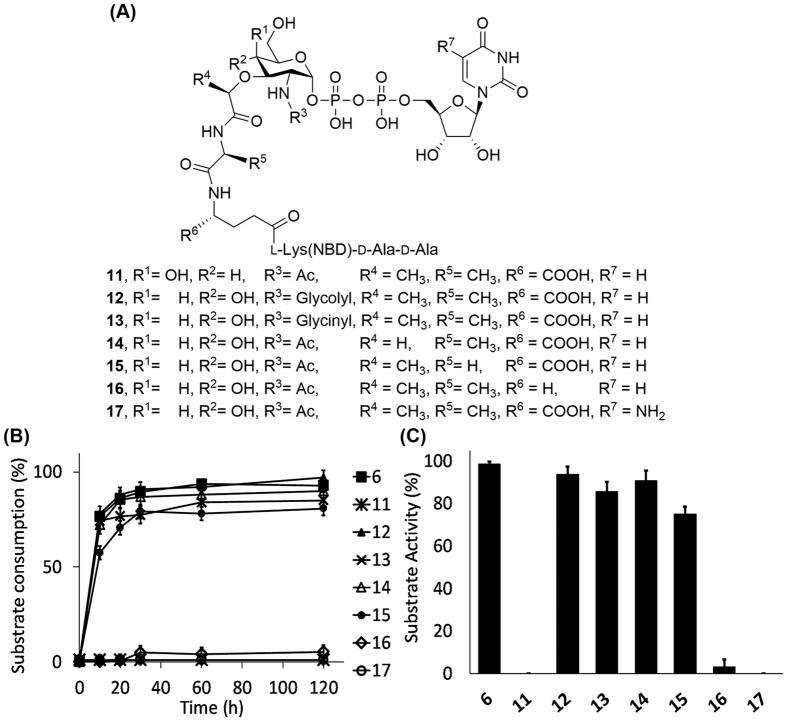
Evaluation of the substrate activity of Park’s nucleotide analogues 6 and 11–17 toward MraY_BS_. (**A**) The chemical structures of synthetic Park’s nucleotide analogues (**11**–**17**) are shown. (**B**) The reactions were analyzed in the HPLC-based MraY functional assay as described in Methods, and the progresses were measured at 0, 10, 20, 30, 60 and 120 min. (**C**) The substrate activity of **6** and **11**–**17** was determined by the substrate consumption after 1 h reaction time. All experiments were repeated in triplicate (Supplementary Figure 4).

Next, more subtle structural changes of Park’s nucleotide **5** were proposed, and the resultant molecules conjugated with a NBD fluorophore on the peptide stem for easy monitoring (Fig. 5A). All analogues except **17** were synthesized in a manner similar to that for **5**. Initial attempts to prepare **17** by coupling of **3** and morpholine-activated 5-amino-uridine-5′-monophosphate in the presence of 1*H*-tetrazole were not successful. Most of the morpholine-activated 5′-NH_2_-UMP was found to degrade into 5′-NH_2_-UMP, and only trace among of product was detected in the reaction mixure^18^. To overcome this problem, the synthetic strategy was re-designed to entail activation of the sugar moiety with the carbonyl diimidazole (CDI) instead of activation of 5-amino-uridine-5′-monophosphate, followed by global deprotection and the NBD labeling^19^. In this way, **17** was obtained in a yield of 31% over four steps (see also Supplementary Methods).

As illustrated in Fig. 5B, both *N*-glycolyl **12**, the natural substrate for mycobacterial MraY (also called MurX), and unnatural *N*-glycinyl **13** had similar substrate activity to **6**, indicating that there are no extra interactions, such as additional hydrogen bonds, to increase the activity between the *N*-substituent moiety on muramic acid of Park’s nucleotide analogues and MraY_BS_^20^. Analogue **14** (R^4^ = H) had similar activity to **6**, suggesting the methyl group on the lactate moiety to be unessential^21^. Likewise, **15** (R^5^ = H) was slightly less active than **6** (about 80% relative activity after 1 h reaction, Fig. 5C)^12^. Surprisely, **16** (R^6^ = H) was found to be a very poor substrate compared to **6** (<10% relative activity after 1 h reaction, Fig. 5C), showing the acid group attached to the Cα of *iso*-d-Glu moiety in Park’s nucleotide to be critical.

**Figure 6:**
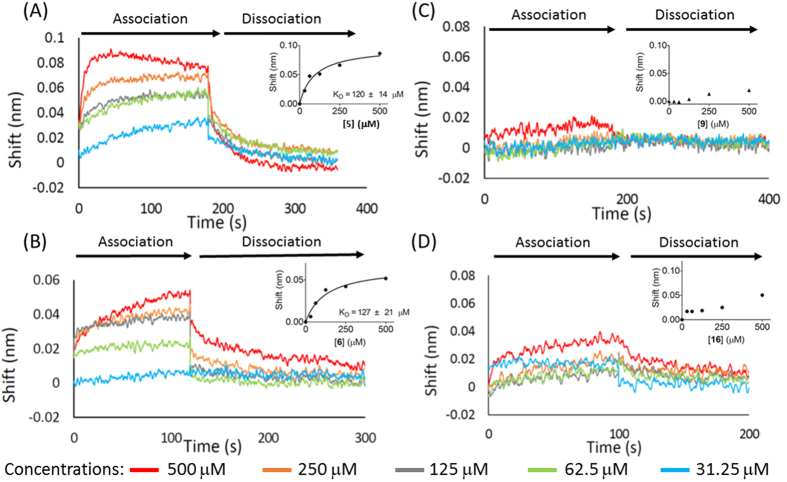
Evaluation of the binding affinity of 5, 6, 9 and 16 toward MraY_BS_. The binding affinity assay was performed by the biolayer interferometry-based binding (BLI) assay as described in Methods. The BLI sensorgrams of (**A**) **5**, (**B**) **6**, (**C**) **9** and (**D**) **16** binding to MraY_BS_ are shown. The dissociation constants (*K*_D_) were obtained by nonlinear regression analysis using the specific binding model with the GraphPad Prism program.

In order to evaluate the effects of peptide moiety of Park’s nucleotide, investigation of the binding affinity of **5**, **6**, **9** and **16** was performed using a biolayer interferometry-based binding (BLI) assay. Initial attempts to perform the MraY_BS_ binding assay in the presence of both substrates (C55P and Park’s nucleotide analog) didn’t work properly because a strong non-specific binding signal was observed; presumably, the hydrophobic part of C55 might mainly contribute this non-specific interaction^22^. To simplify the assay conditions, only Park’s nucleotides were utilized to measure the binding affinity with MraY_BS._ As shown in Fig. 6, compounds **5** and **6** exhibited similar binding affinity with *K*_D_ values of 120 and 127 μM, respectively. This suggests the NBD tag in **6** does not affect the binding affinity with MraY_BS_, and this observation is consistent with the substrate activity result in Fig. 4. Structurally, **9** is the truncated form of **5** (lacking the outermost four amino acids, including *iso*-d-Glu); and **16** is very similar to **6** – the only difference being removal of the acid group attached to the Cα of *iso*-d-glutamic acid (R^6^ = H). However, **9** and **16** showed no proper binding affinity with MraY_BS_ – only a very low binding signal was detected, even at concentrations up to 500 μM. Our results indicate that the acid moiety (R^6^ = COOH) on *iso*-d-Glu of Park’s nucleotide plays an important role for both binding affinity and substrate activity. In addition, **11** (R^1^ = OH/R^2^ = H) and **17** (R^7^ = NH_2_) did not function as substrates, even under extreme reaction conditions, showing the equatorial hydroxyl group at R^2^ position to be critical and the modifications at R^7^ position not tolerated.

### Construction of the molecular model of the Park’s nucleotide- MraY_BS_ complex

**Figure 7:**
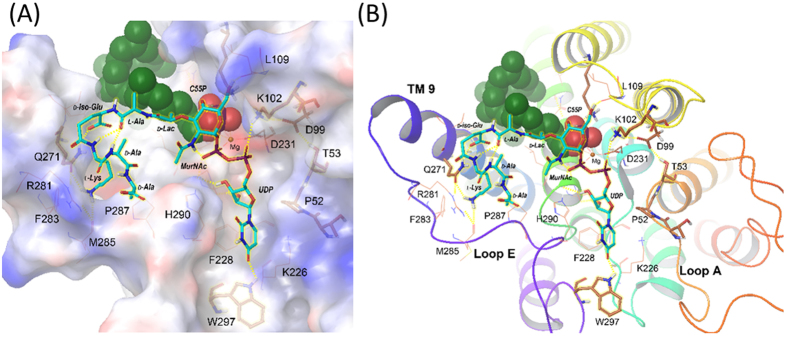
Docked pose of Park’s nucleotide 5 in the MraY_BS_ binding site. (**A**) Surface view of the substrate binding site of MraY_BS_ and the docked ligand poses. (**B**) Cartoon view of the MraY_BS_ activity site and the docked ligand poses. TM refers to transmembrane domain. The carbons of ligand (Park’s nucleotide **5**) are colored in light blue and the carbons of protein are colored in brown. C55P is shown by a ball style representation. Amino acid residues of the protein are labeled in one-letter code; residues of the ligand are labeled by three-letter code.

Based on these preceding results, as well as our mutagenesis (Table 1) and computational modeling studies, a putative Park’s nucleotide binding site on MraY_BS_ is proposed (Fig. 7)^23^,^24^. As illustrated in Fig. 7A, Park’s nucleotide **5** could specifically interact with the W297, K102, and Q271 of MraY, and the phosphate group of C55P. As shown in Table 1, the enzyme activities of four MraY_BS_ mutants, including T53A, K102A, Q271A, and W297A, were significantly decreased, suggesting these residues to be important for enzyme activity. All four mutants had higher *K*_M_ values compared with the wild-type MraY_BS_ (*K*_M_ = 18 μM and NBD-Park’s nucleotide **6** applied as a substrate), showing that the mutations caused a loss of binding affinity. The highly conserved threonine (T53) located on loop A is close to the proposed catalytic pocket (Fig. 7B), and may participate in the enzyme process. In addition, the uracil moiety is embedded in a deep grove, which may interact with W297 on loop E of MraY_BS_. Our substrate specificity and site-directed mutagenesis study strongly suggest that Q271 on MraY_BS_ might interact with the *iso*-d-glutamic acid of Park’s nucleotide through a hydrogen bond to stabilize the peptide chain.

### Discovery of Park’s nucleotide analogues bearing modifications at the uracil 5-position as MraY_BS_ inhibitors

**Figure 8:**
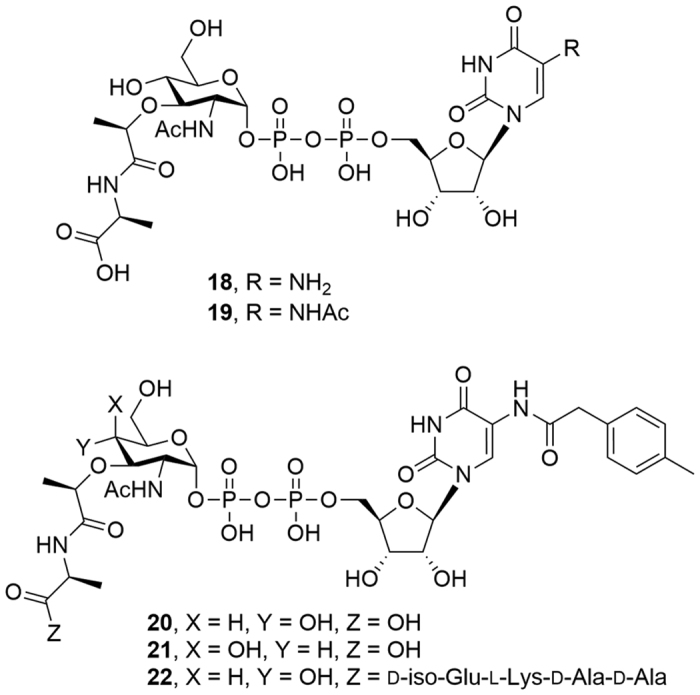
Structures of Park’s nucleotide analogues 18–22.

We were curious whether analogues **11** (modified at R^1^/R^2^) and **17** (modified at R^7^) – neither of which were active substrates – could inhibit the function of MraY_BS_. To further evaluate the role of positions at R^1^, R^2^ and R^7^, we re-designed and synthesized **18**–**21** with a truncated peptide (Fig. 8). The inhibitory activity of each of these was determined using a fluorescent enhancement assay against MraY_BS_, with tunicamycins as reference (Supplementary Figure 5). As shown in Table 2, **18** (R^7^ = NH_2_) had no inhibition activity, but **19** (R^7^ = NHAc) showed weak inhibitory activity (*K*_i_ = 764 μM) against MraY_BS_; and **20**, bearing a *p*-tolylacetamide moiety at R^7^, became a more potent inhibitor (*K*_i_ = 11 μM) –strongly indicating that appropriate *N*-substitution (R^7^) can enhance inhibitory activity. However, **21**, the *C*4-hydroxyl epimer of **20**, was a very poor inhibitor (30% inhibition at 1 mM). This finding implied the *C*4-hydroxyl epimerization of Park’s nucleotide dramatically impairs both the substrate and inhibitory activities. To improve the inhibitory activity, we reinstalled the tetrapeptide moiety on **20** to give **22**, behaving as a competitive inhibitor with the *K*_i_ value of 4 μM toward MraY_BS_, approximately two-fold more potent than tunicamycins (*K*_i_ = 9 μM). Our results indicated that (1) analogues **19**, **20**, and **22** all function as MraY_BS_ inhibitors, even though they all contain a pyrophosphate moiety; and (2) inhibitory potency can be significantly improved through modification of the substituent at the 5-position of the uracil; modification of the oligopeptide moiety can moderately increase the inhibition activity.

To understand the binding contribution derived from structural modifications, **19**–**22** were used to evaluate their binding affinity toward MraY_BS_ in the BLI assay (Table 2 and Supplementary Figure 6). The results are interesting. For example, moderate binding signals were revealed after acetylation of **18**, and the binding affinity of **20** (R = *p*-tolylacetamido group, *K*_D_ = 197 μM) was approximately 1.5 fold stronger than that of **19** (R = acetamido group, *K*_D_ = 281 μM). From the modeled complex structure, both inhibitors could exert an additional H-bond interaction with the K226 in the hydrophobic cleft formed by P52, K226, F228, and W297 (refer to Fig. 7B) to compensate the losing interaction with Q271. Although the binding affinity of **19** and **20** is subject to the modification at the 5-position of the uracil, there is no obvious reason to explain why their inhibition abilities (*K*_i_ values in Table 2) vary by 70 fold. Presumably, the *p*-tolylacetamido substituent at the 5-position of the uracil might further affect the interaction with MraY_BS_ by inducing an enzyme conformation change – a detailed molecular dynamic simulation of Park’s nucleotide and inhibitors toward MraY_BS_ remains to be performed. Compound **21** had no sufficient binding interactions with MraY_BS_ even at the concentration up to 250 μM, emphasizing that the orientation of the *C*4-hydroxyl group of muramic acid plays an essential role in molecule-enzyme recognition. Notably, the binding affinity of **22** (*K*_D_ = 86 μM) was approximately 2.3 fold stronger than that of **20**, which can be attributed to the contribution of the tetrapeptide moiety.

The antibacterial activities of **20** and **22** were also investigated and the minimal inhibitory concentrations (MIC) against *S. aureus* and *B. subtilis* were determined using standard-broth dilution methods^25^. Unfortunately, both **20** and **22** showed no antibacterial activity, even at a high concentration of 200 μM. It may be because the compounds containing the highly charged pyrophosphate moiety were difficult to penetrate the bacterial cytoplasmic membrane^26^. In order to improve the antibacterial activity, finding a surrogate to replace the pyrophosphate moiety on **22** remains to be explored.

## Conclusions

**Figure 9:**
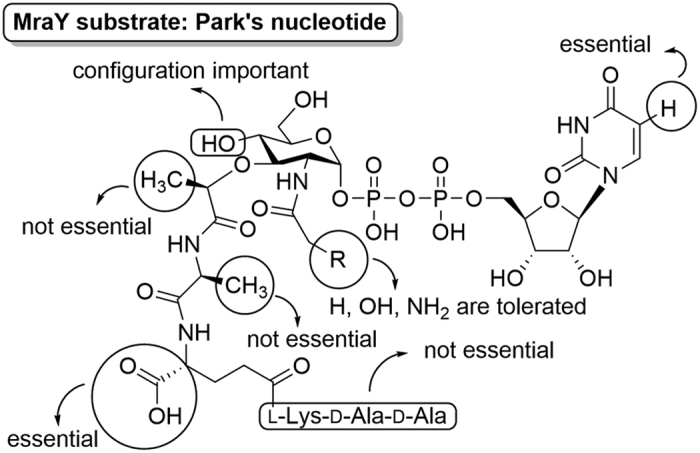
Summary of substrate specificity of Park’s nucleotide toward MraY.

A series of Park’s nucleotide analogues with modifications at the peptide, muramic acid, and nucleotide moieties has been designed and synthesized, and their MraY_BS_ substrate activity and specificity were evaluated. Our results led to several important findings: (1) the first two amino acids (l-alanine-*iso*-d-glutamic acid) of the oligopeptide chain are essential for MraY_BS_ recognition; (2) the configuration of the *C*4-OH on muramic acid is important for MraY_BS_ substrate specificity; and (3) modifications at the 5-position of the uracil dramatically impair the substrate activity (Fig. 9). Also, the substrate specificity data together with mutagenesis and computational modeling studies allowed us to infer a putative Park’s nucleotide binding site on MraY_BS_.

Unexpectedly, analogues bearing modifications at the 5-position of the uracil were found to be MraY_BS_ inhibitors though these molecules contain a pyrophosphate moiety. Of these, an unoptimized inhibitor **22** (*K*_i_ = 4 μM) was found to be roughly twice as potent as tunicamycins (*K*_i_ = 9 μM) against MraY_BS_, the first example of Park’s nucleotide-based inhibitors. However, our results not only allow us to infer the minimal structure requirements of Park’s nucleotide as a MraY substrate, but also illuminate a new direction for MraY inhibitor design. And more generally, the concrete nature of these conclusions validate our strategy of systematic substrate structure modifications for the elucidation of enzyme binding site mapping, for membrane proteins without available co-crystal structures. Investigation of the MraY mechanisms, and development of more potent inhibitors with *in vivo* antibacterial activity are currently ongoing in our laboratory.

## Methods

### General

All chemicals were obtained from commercial suppliers and used without further purification. All solvents were anhydrous grade unless indicated otherwise. All non-aqueous reactions were performed in oven-dried glassware under a slight positive pressure of argon unless otherwise noted. Reactions were magnetically stirred and monitored by thin-layer chromatography on silica gel. Flash chromatography was performed on silica gel of 40–63 μm particle size. Concentration refers to rotary evaporation. Yields are reported for spectroscopically pure compounds. NMR spectra were recorded on dilute solutions in D_2_O, CDCl_3_ and CD_3_OD on Bruker AVANCE 600 at ambient temperature. Chemical shifts are given in *δ* values and coupling constants *J* are given in Hz. The splitting patterns are reported as s (singlet), d (doublet), t (triplet), q (quartet), m (multiplet), and dd (double of doublets). High resolution ESI mass spectra were recorded on a Bruker Daltonics spectrometer. Compound **7**–**22** were prepared as described in supplementary Methods. Tunicamycins were purchased from Sigma-Aldrich (St. Louis, MO).

### Chemistry

#### Compound 2

A mixture of **1** (200 mg, 0.31 mmol) and 20% Pd(OH)_2_/C (20 mg) in THF (10 mL) was stirred at RT for 24 h under a hydrogen atmosphere. The reaction mixture was filtered through a pad of celite and the filtrate was concentrated to give a crude intermediate as white solid. The intermediate was used directly without further purification. The solution of intermediate, 1*H*-tetrazole (43 mg, 0.62 mmol) and dibenzyl (*N*,*N*-diisopropyl) phosphoramidite (213 mg, 0.62 mmol) in CH_2_Cl_2_/ACN (10 mL, v/v = 1:1) was stirred at 0 °C for 2 h. The reaction was then cooled to −40 °C and treated with *tert*-butylhydroperoxide (0.6 mL, 6.0 mmol). The reaction was allowed to warm to RT over a period of 1 h. The mixture was diluted with CH_2_Cl_2_ (50 mL), and extract with water (20 mL × 2). The organic layers were collected, dried over MgSO_4_, concentrated, and purified by cc (EtOAc/CH_2_Cl_2_ = 1:2, silica gel) to afford **2** as a colorless oil (177 mg, 0.22 mmol, 71% over three steps). ^1^H NMR (600 MHz, CDCl_3_): *δ* 7.26–7.31 (m, 10 H), 6.72 (d, 1 H, *J* = 7.2 Hz), 6.58 (d, 1 H, *J* = 9.0 Hz), 5.61 (dd, 1 H, *J* = 5.4 and 6.0 Hz), 4.96–5.05 (m, 5 H), 4.31–4.34 (m, 1 H), 4.23–4.26 (m, 1 H), 4.11–4.14 (m, 2 H), 4.02 (dd, 1 H, *J* = 4.8 and 13.2 Hz), 3.84–3.92 (m, 3 H), 3.49 (dd, 1 H, *J* = 9.6 and 10.2 Hz), 2.01 (s, 3 H), 1.94 (s, 3 H), 1.71 (s, 3 H), 1.35 (d, 3 H, *J* = 7.2 Hz), 1.25 (d, 3 H, *J* = 6.6 Hz), 0.91–0.93 (m, 2 H), −0.03 (s, 9 H); ^13^C NMR (150 MHz, CDCl_3_): δ 172.4, 172.0, 170.7, 170.6, 169.0, 135.2, 135.0, 128.9 (×2), 128.7 (×4), 129.0 (×2), 128.0 (×2), 96.7, 78.1, 76.6, 70.1, 69.9 (×2), 68.8, 63.8, 61.4, 53.0, 48.1, 22.8, 20.7, 20.6, 18.8, 17.6, 17.1, −1.59 (×3); HRMS calcd for [C_37_H_53_N_2_O_14_PSi + H]^+^ 809.3081, found 809.3045.

#### Compound 3

A mixture of **2** (100.0 mg, 0.12 mmol) and 1.0 M TBAF in THF (0.24 mL, 0.24 mmol) in THF (5 mL) was stirred at RT for 2 h. The reaction mixture was concentrated, and the residue was extracted with EtOAc (50 mL), washed with 1.0 N HCl_(aq)_ (50 mL × 2) and water (50 mL × 2). The combined organic layers were dried over MgSO_4_, and concentrated to afford the monosaccharide intermediate as a colorless oil. The sugar intermediate and PyBOP (124.0 mg, 0.24 mmol) in CH_2_Cl_2_ (5 mL) were stirred at 0 °C for 5 min. To the above mixture, a solution of H-d-Glu(OMe)-l-Lys(TFA)-d-Ala-d-Ala(OMe) (129 mg, 0.24 mmol), and DIEA (104.3 μL, 0.6 mmol) in THF (5 mL) were added. The reaction mixture was stirred at RT for 30 min. The reaction solvent was removed and the residue was extracted with CH_2_Cl_2_ (50 mL), 1.0 N HCl_(aq)_ (50 mL × 2) and water (50 mL × 2). The combined organic layers were dried over MgSO_4_, filtered, concentrated and purified by cc (CH_2_Cl_2_/MeOH/H_2_O = 60:25:4, silica gel) to give benzyl protected monophosphate intermediate as colorless oil. A mixture of this intermediate (125 mg, 0.1 mmol) and 20% Pd(OH)_2_/C (10 mg) in MeOH (8 mL) was stirred for 1 h under a hydrogen atmosphere. The reaction mixture was filtered through a pad of celite, and the filtrate was concentrated and purified by cc (CHCl_3_/MeOH/H_2_O = 60:25:4, silica gel) to give **3** as a colorless oil (84 mg, 0.08 mmol, 67% over three steps). ^1^H NMR (600 MHz, CD_3_OD): *δ* 5.45 (dd, 1 H, *J* = 3.0 and 6.6 Hz), 5.06 (t, 1 H, *J* = 9.6 Hz), 4.36–4.40 (m, 3 H), 4.16–4.28 (m, 6 H), 4.10 (dd, 1 H, *J* = 1.8 and 12.0 Hz), 3.86 (t, 1 H, *J* = 9.6 Hz), 3.71 (s, 3 H), 3.69 (s, 3 H), 2.28–2.30 (m, 2 H), 2.20–2.23 (m, 1 H), 2.09 (s, 3 H), 2.04 (s, 3 H), 1.95 (s, 3 H), 1.88–2.01 (m, 2 H), 1.77–1.80 (m, 1 H), 1.70–1.72 (m, 1 H), 1.59–1.61 (m, 2 H), 1.42 (d, 3 H, *J* = 6.6 Hz), 1.38 (d, 3 H, *J* = 7.7 Hz), 1.31 (d, 3 H, *J* = 6.6 Hz), 1.28–1.45 (m, 6 H); ^13^C NMR (150 MHz, CD_3_OD): *δ* 173.6, 173.5, 173.4, 173.1 (×2), 172.1, 171.9, 171.2, 170.8, 170.3, 157.5, 115.2, 93.9, 78.0, 77.8, 69.2, 68.5, 61.8, 54.4, 54.2, 53.8, 51.5, 51.4, 51.3, 49.4, 48.9, 39.0, 30.8, 30.6, 28.1, 26.7, 22.7, 21.7, 19.6, 19.3, 17.8, 16.6, 16.3, 15.9; HRMS calcd for [C_39_H_61_F_3_N_7_O_21_P + Na]^+^ 1074.3502, found 1074.3518.

#### Compound 4

A mixture of **2** (100 mg, 0.12 mmol) and 20% Pd(OH)_2_/C (10 mg) in MeOH (8 mL) was stirred for 1 h under a hydrogen atmosphere. The reaction mixture was filtered through a pad of celite, and the filtrate was concentrated and purified by cc (CHCl_3_/MeOH/H_2_O = 60:25:4, silica gel) to give **4** as white solid (57 mg, 0.09 mmol, 75%). ^1^H NMR (600 MHz, CD_3_OD): *δ* 5.08 (t, 1 H, *J* = 9.6 Hz), 4.16–4.32 (m, 8 H), 4.10 (d, 1 H, *J* = 12.6 Hz), 3.86 (t, 1 H, *J* = 9.6 Hz), 2.11 (s, 3 H), 2.06 (s, 3 H), 1.95 (s, 3 H), 1.40 (d, 3 H, *J* = 7.2 Hz), 1.31 (d, 3 H, *J* = 6.6 Hz), 1.01 (m, 2 H), 0.06 (s, 9 H); ^13^C NMR (150 MHz, CD_3_OD): δ 175.2, 174.1, 173.8, 172.8, 171.8, 95.3, 79.3, 79.0, 70.9, 70.1, 64.8 (×2), 63.4, 55.5, 23.3, 21.2, 21.0, 19.5, 18.3, 17.6, −1.22 (×3). HRMS calcd for [C_23_H_41_N_2_O_14_PSi−H]^−^ 627.1981, found 627.1996.

#### Compound 5

A mixture of **3** (84.0 mg, 0.08 mmol), UMP-morpholine-*N*,*N′*-dicyclohexyl-carboxamidine salt (80.0 mg, 0.12 mmol), 1*H-*tetrazole (8.4 mg, 0.12 mmol) and 4 Å molecular sieve in anhydrous pyridine was stirred at RT for 24 h under argan. The reaction was concentrated and purified by cc (CHCl_3_/MeOH/H_2_O = 60:25:4, silica gel) to give a pyrophosphate intermediate. The intermediate was dissolved in a solution of 1.0 M LiOH_(aq)_/MeOH (2 mL, v/v = 1:1) and stirred at RT for the global deprotection. After stirring for 4 h, the reaction was neutralized by 1.0 N HCl_(aq)_, concentrated, and purified by cc (iPrOH/NH_4_OH_(aq)_ = 2/1, silica gel) to give **5** as white solid (32 mg, 0.027 mmol, 35% over two steps). ^1^H NMR (600 MHz, D_2_O): *δ* 7.94 (d, 1 H, *J* = 8.4 Hz), 5.94–5.96 (m, 2 H), 5.45 (dd, 1 H, *J* = 3.6 and 7.2 Hz), 4.03‒4.36 (m, 15 H), 3.91–3.94 (m, 1 H), 3.80–3.87 (m, 2 H), 3.77 (t, 1 H, *J* = 10.2 Hz), 3.62 (dd, 1 H, *J* = 9.0 and 10.2 Hz), 2.98 (m, 2 H), 2.28 (t, 1 H, *J* = 7.8 Hz), 2.12–2.14 (m, 1 H), 1.98 (s, 3 H), 1.65–1.89 (m, 5 H), 1.31–1.44 (m, 12 H). HRMS calcd for [C_40_H_65_N_9_O_26_P_2_ + H]^+^ 1150.3589, found 1150.3644.

#### Compound 6

A mixture of Park’s nucleotide **5** (10.0 mg, 8.7 μmol) and NBD-X-OSu (3.3 mg, 8.6 μmol) in a solution of DMF/sat. NaHCO_3(aq)_ (2 mL, v/v = 1:1) was stirred at RT for 2 h. The solvent was removed and the reaction mixture was purified by cc (iPrOH/NH_4_OH = 2/1, silica gel) to give a fluorescent product. The compound was further purified by a semi-preparative reverse-phase HPLC (ZORBAX RX-C18 column, 5 μm, 9.4 × 250 mm) with gradient elution of 0.25 M NH_4_HCO_3(aq)_/15% MeOH in water (100:0 to 0:100) at a flow rate of 1 mL/min over 45 min, to give compound **6** as a reddish brown solid (11.0 mg, 7.7 μmol, 88%). The purity of **6** is higher than 95% by analytical anion-exchange HPLC (Supplementary Figure 7). HRMS calcd for [C_52_H_77_N_13_O_30_P_2_ + H]^+^ 1426.4447, found 1426.3308.

### Biology

#### HPLC-based MraY functional assay

The purified MraY from *Bacillus subtilis* was prepared as described in our previous reports^16^. For **5** and **7**–**10**, the reaction mixture containing MraY_BS_ (10 μg/mL), C_55_P (200 μM) in 40 μL reaction buffer (30 mM Tris, 10 mM MgCl_2_, 10 mM NaCl, 0.1 mM Tween-20, 2.5% DMSO, pH 8.0) were pre-incubated at 37 °C for 15 min. The reactions were initiated by the addition of Park’s nucleotide analogues (10 μM) and incubated at 37 °C for 1 h. The reaction mixtures were added by uridine (0.01 mg/mL) as the internal standard and heated to 100 °C to stop the transferring reaction. The samples were analyzed by RP-C18 HPLC with gradient elution of 0.25 M NH_4_HCO_3(aq)_/15% MeOH in water (100:0 to 0:100) at a flow rate of 1 mL/min over 45 min, and the peaks of substrates were monitored at UV 260 nm. For **6** and **11**–**17**, the reaction mixture containing MraY_BS_ (10 μg/mL), C_55_P (200 μM), 6-(7-Nitro-2,1,3-benzoxadiazol-4-ylamino)hexanoic acid (5 μM, internal standard) in 10 μL reaction buffer (30 mM Tris, 10 mM MgCl_2_, 10 mM NaCl, 0.1 mM Tween-20, 2.5% DMSO, pH 8.0) were pre-incubated at 37 °C for 15 min. The reactions were initiated by the addition of NBD-Park’s nucleotide analogues (10 μM) and incubated at 37 °C for 1 h. The reaction mixtures were heated to 100 °C to stop the transferring reaction, and samples were analyzed by an anion-exchange column (SAX1, Supelco Co., 5 μm, 4.6 × 250 mm) with a linear gradient elution of NH_4_OAc (20 mM to 1 M in MeOH) at a flow rate of 1.0 mL/min over 30 min. The fluorescent substrates were monitored with λ_ex_ 466 nm/λ_em_ 535 nm by fluorescence detector. In an extreme reaction condition, which is performed in reaction buffer (30 mM Tris, 10 mM MgCl_2_, 10 mM NaCl, 0.1 mM Tween 20, 2.5% DMSO, pH 8.0) containing Park’s nucleotides (10 μM), MraY_BS_ (40 μg/mL) and C55P (1 mM) at 37 °C for 24 h, compounds **11**, **17** and **22** were confirmed to not be a substrate.

#### Biolayer interferometry-based (BLI) binding assay

MraY_BS_ binding was measured in a biolayer interferometry-based binding assay on Octet Red96^®^ instrument (ForteBio, Inc.). The biotinylation of MraY_BS_ was performed with using EZ-Link^®^ Sulfo-NHS-LC-LC-Biotin Kit (Thermo Fisher Scientific, Inc.). In general, MraY_BS_ (1.7 mg/mL) in 50 μL reaction buffer (0.01 M HEPES pH 7.4, 0.15 M NaCl, 0.005% v/v Surfactant P20), was added by the sulfo-NHS-LC-LC-biotin reagent (1 mM, 1 μL). After incubating for 30 minutes at room temperature, the biotinylated-MraY_BS_ was purified by a Zeba^TM^ Spin Desalting Columns, 7K MWCO (Thermo Fisher Scientific, Inc.), and the buffer was changed to the binding buffer (30 mM Tris, 10 mM MgCl_2_, 10 mM NaCl, 0.1 mM Tween-20, pH 8.0) during the purification. Comparing to the non-biotinylated-MraY_BS,_ the biotinylated-MraY_BS_ remains 62% activity measured by the HPLC-based MraY functional assay in 1 h reaction. The biotinylated MraY_BS_ was then immobilized to Super Streptavidin (SSA) Octet tips (ForteBio, Inc.) for reaching a fixed signal of 3 nm. Before the assay starting, the MraY_BS_-labeled SSA sensors were pre-soaked in binding buffer for 30 min. The association of Park’s analogues with MraY_BS_ were measured by incubating MraY_BS_-labeled SSA sensors in binding buffer containing various concentrations of samples in Octet Red system. The dissociation was monitored by moving the ligand biosenors from the analyte solution to binding buffer. The dissociation constants (*K*_D_) were obtained by nonlinear regression analysis using the specific binding model with the GraphPad Prism program (GraphPad Software, San Diego, CA).

## Additional Information

**How to cite this article**: Chen, K.-T. *et al*. Structural Investigation of Park’s Nucleotide on Bacterial Translocase MraY: Discovery of Unexpected MraY Inhibitors. *Sci. Rep.* **6**, 31579; doi: 10.1038/srep31579 (2016).

## Supplementary Material

Supplementary Information

## Figures and Tables

**Figure 1:**
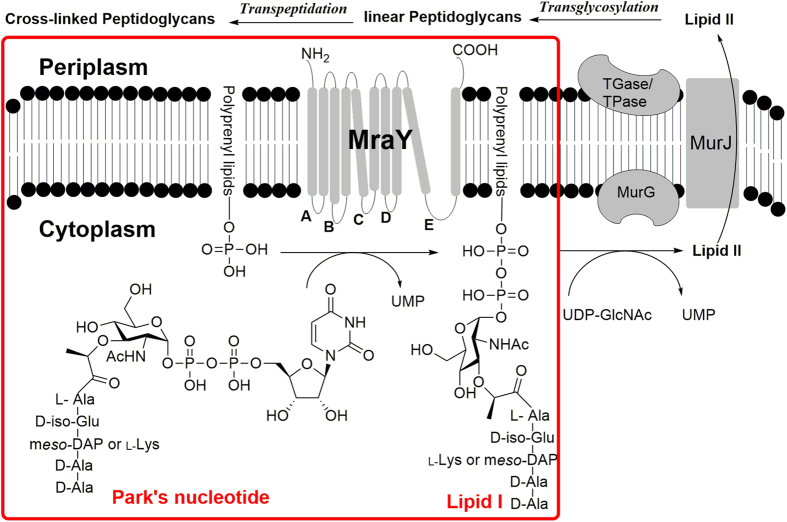
Role of MraY in bacterial peptidoglycan biosynthesis and the chemical structures of Park’s nucleotide and Lipid I.

**Figure 2:**
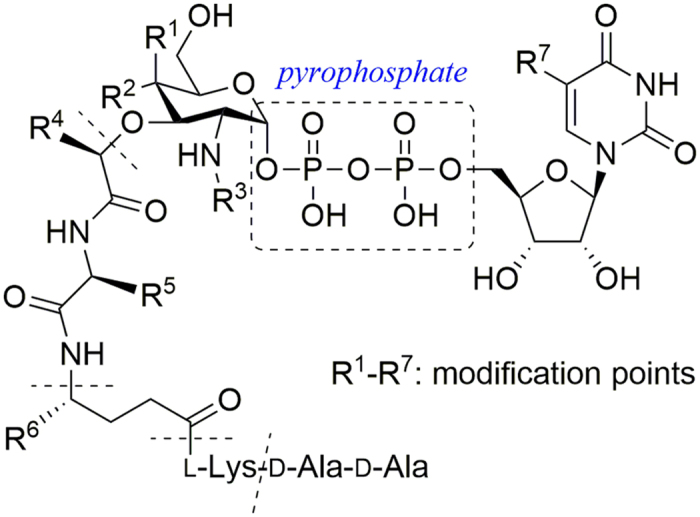
Structures of Park’s nucleotide analogues with proposed modified positions.

**Table 1:** Evaluation of kinetic parameters of mutant MraYs.

Proteins	*K*_M_ (μM)^a^	V_max_ (FLU/min)^a^	Relative activity (%)^b^
WT	18 ± 1	18 ± 0.8	100
T51A	24 ± 2	5 ± 0.2	60
T53A	70 ± 11	7 ± 0.8	18
K102A	49 ± 9	5 ± 0.2	33
K226A	24 ± 3	23 ± 4.7	100
F228A	18 ± 2	10 ± 1.5	98
Q271A	72 ± 11	6 ± 1.7	16
R281A	20 ± 1	10 ± 1.4	81
W297A	63 ± 9	4 ± 0.4	17

^a^Kinetic parameters were determined by fluorescent enhancement assay with using **6** as the substrate. FLU: fluorescent units^27^.

^b^The assay was determined by HPLC-based MraY functional assay with using **6** as the substrate. Relative activity was determined by monitoring the consumption of **6** in one-hour reaction.

**Table 2:** Evaluation of the inhibitory activity and the binding affinity of Park’s nucleotide derivatives (18–22) toward MraY_BS_.

Entry	Compounds	K_i_ (μM)^a^	Inhibition Mode^a^	K_D_ (μM)^b^
1	**18**^c^	**—**d	**—**d	Not determined^c^
2	**19**	764 ± 127	Competitive	281 ± 97
3	**20**	11 ± 3	Competitive	197 ± 47
4	**21**^e^	**—**d	**—**d	NB^f^
5	**22**^g^	4 ± 1	Competitive	86 ± 12
6	**Tunicamycins**	9 ± 1	Competitive	93 ± 16

^a^The *K*_i_ and inhibition mode were determined by fluorescent enhancement assay.

^b^The dissociation constants (*K*_D_) were determined by BLI assay.

^c^No inhibitory activity was observed at 1 mM for **18**. The *K*_D_ value of **18** is not determined.

^d^The *K*_i_ and inhibition mode cannot be measured due to the low inhibitory activity.

^e^Only 30% inhibition was observed at 1 mM for **21**.

^f^NB refers to no significant binding signal at 250 μM.

^g^Compound **22** was not a MraY_BS_ substrate even under extreme conditions.

## References

[b1] Levy S. B. . Antibacterial resistance worldwide: causes, challenges and responses. Nat Med 10, S122–S129 (2004). 15577930 10.1038/nm1145

[b2] Ostash B. . Bacterial transglycosylase inhibitors. Curr Opin Chem Biol 9, 459–466 (2005). 16118062 10.1016/j.cbpa.2005.08.014

[b3] Boyle D. S. & Donachie. W. D. mraY is an essential gene for cell growth in Escherichia coli. J Bacteriol 180, 6429–6432 (1998). 9829961 10.1128/jb.180.23.6429-6432.1998 PMC107738

[b4] Bugg T. D. . Phospho-MurNAc-pentapeptide translocase (MraY) as a target for antibacterial agents and antibacterial proteins. Infect Disord Drug Targets 6, 85–106 (2006). 16789873 10.2174/187152606784112128

[b5] Zawadzke L. E. . Targeting the MraY and MurG bacterial enzymes for antimicrobial therapeutic intervention. Anal Biochem 314, 243–252 (2003). 12654311 10.1016/s0003-2697(02)00622-x

[b6] Winn M. . Antimicrobial nucleoside antibiotics targeting cell wall assembly: recent advances in structure-function studies and nucleoside biosynthesis. Nat Prod Rep 27, 279–304 (2010). 20111805 10.1039/b816215h

[b7] Price N. P. . Biosynthesis of the tunicamycins: a review. J Antibiot 60, 485–491 (2007). 17827659 10.1038/ja.2007.62

[b8] Dini C. . MraY Inhibitors as Novel Antibacterial Agents. Curr Top Med Chem 5, 1221–1236 (2005). 16305528 10.2174/156802605774463042

[b9] Takahashi Y. . Novel semisynthetic antibiotics from caprazamycins A-G: caprazene derivatives and their antibacterial activity. J Antibiot 66, 171–178 (2013). 23532021 10.1038/ja.2013.9

[b10] Kimura K. . Recent advances in antimicrobial nucleoside antibiotics targeting cell wall biosynthesis. Nat Prod Rep 20, 252–273 (2003). 12735700 10.1039/b202149h

[b11] Chung B. C. . Crystal structure of MraY, an essential membrane enzyme for bacterial cell wall synthesis. Science 341, 1012–1016 (2013). 23990562 10.1126/science.1236501 PMC3906829

[b12] Hammes W. P. . On the specificity of phospho-*N*-acetylmuramyl-pentapeptide translocase. The peptide subunit of uridine diphosphate-N-actylmuramyl-pentapeptide. J Biol Chem 249, 3140–3150 (1974). 4208473

[b13] Egan A. J. . Activities and regulation of peptidoglycan synthases. Philos Trans R Soc Lond B Biol Sci 370, 20150031 (2015). 26370943 10.1098/rstb.2015.0031 PMC4632607

[b14] Breukink E. . Lipid II is an intrinsic component of the pore induced by nisin in bacterial membranes. J Biol Chem 278, 19898–19903 (2003). 12663672 10.1074/jbc.M301463200

[b15] Chen K. T. . Rapid preparation of mycobacterium *N*-glycolyl Lipid I and Lipid II derivatives: a biocatalytic approach. Chem Eur J 19, 834–838 (2013). 23229320 10.1002/chem.201203251

[b16] Huang L. Y. . Enzymatic synthesis of lipid II and analogues. Angew Chem Int Ed Engl 53, 8060–8065 (2014). 24990652 10.1002/anie.201402313

[b17] Liu C. Y. . Synthesis and Evaluation of a New Fluorescent Transglycosylase Substrate: Lipid II-Based Molecule Possessing a Dansyl-C20 Polyprenyl Moiety. Org Lett 12, 1608–1611 (2010). 20187630 10.1021/ol100338v

[b18] Tedaldi L. M. . Optimised chemical synthesis of 5-substituted UDP-sugars and their evaluation as glycosyltransferase inhibitors. Carbohydr Res 364, 22–27 (2012). 23147042 10.1016/j.carres.2012.10.009

[b19] Meng F. C. . Total synthesis of polyprenyl *N*-glycolyl lipid II as a mycobacterial transglycosylase substrate. Org Lett 13, 5306–5309 (2011). 21913698 10.1021/ol2021687

[b20] Siricilla S. . Biosynthesis of a water-soluble lipid I analogue and a convenient assay for translocase I. Anal Biochem 461, 36–45 (2014). 24939461 10.1016/j.ab.2014.05.018 PMC4296562

[b21] Shih H. W. . Effect of the Peptide Moiety of Lipid II on Bacterial Transglycosylase. Angew. Chem Int Ed Engl 51, 10123–10126 (2012). 22952114 10.1002/anie.201204038

[b22] Fernandez-Vidal M., White S. H. & Ladokhin. A. S. Membrane partitioning: “classical” and “nonclassical” hydrophobic effects. J Membr Biol 239, 5–14 (2011). 21140141 10.1007/s00232-010-9321-y PMC3030945

[b23] Al-Dabbagh B. . Active site mapping of MraY, a member of the polyprenyl-phosphate N-acetylhexosamine 1-phosphate transferase superfamily, catalyzing the first membrane step of peptidoglycan biosynthesis. Biochemistry 47, 8919–8928 (2008). 18672909 10.1021/bi8006274

[b24] Banks J. L. . Integrated Modeling Program, Applied Chemical Theory (IMPACT). J Comput Chem 26, 1752–1780 (2005). 16211539 10.1002/jcc.20292 PMC2742605

[b25] Huang S. H. . New continuous fluorometric assay for bacterial transglycosylase using Forster resonance energy transfer. J Am Chem Soc 135, 17078–17089 (2013). 24131464 10.1021/ja407985m

[b26] Wang R. . A search for pyrophosphate mimics for the development of substrates and inhibitors of glycosyltransferases. Bioorg Med Chem 5, 661–672 (1997). 9158864 10.1016/s0968-0896(97)00005-9

[b27] Brandish P. E. . Slow binding inhibition of phospho-*N*-acetylmuramyl-pentapeptide-translocase (Escherichia coli) by mureidomycin A. J Biol Chem 271, 7609–7614 (1996). 8631795 10.1074/jbc.271.13.7609

