# Supplementary material for: Structural Investigation of Park’s Nucleotide on Bacterial Translocase MraY: Discovery of Unexpected MraY Inhibitors
Source: Sci Rep. 2016 Aug 17;6:31579. doi: 10.1038/srep31579 (PMC4987650; doi:10.1038/srep31579)
Supplement: Supplementary Information [file srep31579-s1.pdf]

Structural Investigation of Park's Nucleotide on Bacterial Translocase MraY: Discovery of Unexpected MraY Inhibitors

Supplementary Information

Kuo-Ting Chen, Po-Ting Chen, Cheng-Kun Lin, Lin-Ya Huang, Chia-Ming Hu, Yi-Fan Chang, Hua-Ting Hsu, Ting-Jen R. Cheng, Ying-Ta Wu and Wei-Chieh Cheng

Genomics Research Center, Academia Sinica, No. 128 Academia Road, Section 2, Nankang District, Taipei, 11529, Taiwan

	Contents	Pages
**Supplementary Methods**	Abbreviations	S1
	General	S1
	Chemistry	S1
	Biology	S5
**Supplementary Tables**	**Supplementary table 1.** Substrate activity study of polyprenol phosphates toward $MraY_{BS}$.	S7
**Supplementary Figures**	**Supplementary figure 1.** Substrate utilization of **6** in HPLC-based MraY functional assay.	S8
	**Supplementary figure 2.** Substrate utilization of **5** in HPLC-based MraY functional assay.	S9
	**Supplementary figure 3.** Substrate utilization of **7-10** in HPLC-based MraY functional assay.	S10
	**Supplementary figure 4.** Substrate utilization of **6** and **11-17** in HPLC-based MraY functional assay.	S11
	**Supplementary figure 5.** Competitive inhibition of **19**, **20**, **22** and tunicamycins against $MraY_{BS}$ activity.	S12
	**Supplementary figure 6.** Evaluation of the binding affinity of Park's analogues (**19-22**) and tunicamycins toward $MraY_{BS}$.	S13
	**Supplementary figure 7**. The purity of NBD-Park's nucleotide analogues **6**, **11**, **14**, **15**, **16** and **17**.	S14
	**Supplementary figure 8.** ^{1}H-NMR Spectra of compound **1** (600 MHz, $CDCl_3$)	S15
	**Supplementary figure 9.** ^{13}C-NMR Spectra of compound **1** (150 MHz, $CDCl_3$)	S15
	**Supplementary figure 10.** ^{1}H-NMR Spectra of compound **2** (600 MHz, $CDCl_3$)	S16
	**Supplementary figure 11.** ^{13}C-NMR Spectra of compound **2** (150 MHz, $CDCl_3$)	S16
	**Supplementary figure 12.** ^{1}H-NMR Spectra of compound **3** (600 MHz, CD_3OD)	S17
	**Supplementary figure 13.** ^{13}C-NMR Spectra of compound **3** (150 MHz, CD_3OD)	S17
	**Supplementary figure 14.** ^{1}H-NMR Spectra of compound **4** (600 MHz, CD_3OD)	S18

	**Supplementary figure 15.** ^{13}C-NMR Spectra of compound **4** (150 MHz, CD_3OD)	S18
	**Supplementary figure 16.** ^{1}H-NMR Spectra of compound **5** (600 MHz, D_2O)	S19
	**Supplementary figure 17.** ^{13}C-NMR Spectra of compound **5** (150 MHz, D_2O)	S19
	**Supplementary figure 18.** ^{1}H-NMR Spectra of compound **7** (600 MHz, D_2O)	S20
	**Supplementary figure 19.** ^{13}C-NMR Spectra of compound **7** (150 MHz, D_2O)	S20
	**Supplementary figure 20.** ^{1}H-NMR Spectra of compound **8** (600 MHz, D_2O)	S21
	**Supplementary figure 21.** ^{13}C-NMR Spectra of compound **8** (150 MHz, D_2O)	S21
	**Supplementary figure 22.** ^{1}H-NMR Spectra of compound **9** (600 MHz, D_2O)	S22
	**Supplementary figure 23.** ^{13}C-NMR Spectra of compound **9** (150 MHz, D_2O)	S22
	**Supplementary figure 24.** ^{1}H-NMR Spectra of compound **19** (600 MHz, D_2O)	S23
	**Supplementary figure 25.** ^{13}C-NMR Spectra of compound **19** (150 MHz, D_2O)	S23
	**Supplementary figure 26.** ^{1}H-NMR Spectra of compound **20** (600 MHz, D_2O)	S24
	**Supplementary figure 27.** ^{13}C-NMR Spectra of compound **20** (150 MHz, D_2O)	S24
	**Supplementary figure 28.** ^{1}H-NMR Spectra of compound **21** (600 MHz, D_2O)	S25
	**Supplementary figure 29.** ^{13}C-NMR Spectra of compound **21** (150 MHz, D_2O)	S25
	**Supplementary figure 30.** ^{1}H-NMR Spectra of compound **22** (600 MHz, D_2O)	S26
**References**		S27

Supplementary Methods

Abbreviations. (1) RT: room temperature; (2) cc: column chromatography; (3) Et_3N: trimethylamine; (4) TBAF: *tetra-N*-butylammonium fluoride; (5) PyBOP: benzotriazol-1-yl-oxytripyrrolidinophosphonium hexafluorophosphate; (6) HPLC: high performance liquid chromatography; (7) DIEA: *N*,*N*-diisopropylethylamine; (8) NBD-X-OSu: succinimidyl 6-(*N*-(7-nitrobenz-2-oxa-1,3-diazol-4-yl)amino)hexanoate; (9) CDI: carbonyldiimidazole.

General. All chemicals were obtained from commercial suppliers and used without further purification. All solvents were anhydrous grade unless indicated otherwise. All non-aqueous reactions were performed in oven-dried glassware under a slight positive pressure of argon unless otherwise noted. Reactions were magnetically stirred and monitored by thin-layer chromatography on silica gel. Flash chromatography was performed on silica gel of 40–63 μm particle size. Concentration refers to rotary evaporation. Yields are reported for spectroscopically pure compounds. NMR spectra were recorded on dilute solutions in D_2O, $CDCl_3$ and CD_3OD on Bruker AVANCE 600 at ambient temperature. Chemical shifts are given in δ values and coupling constants J are given in Hz. The splitting patterns are reported as s (singlet), d (doublet), t (triplet), q (quartet), m (multiplet), and dd (double of doublets). High resolution ESI mass spectra were recorded on a Bruker Daltonics spectrometer. Compound **S1** was prepared as described in our previous report.[1] Compound **10** (UDP-GlcNAc) and tunicamycins were purchased from Sigma-Aldrich (St. Louis, MO). Compound **12** and **13** were prepared as described in our previous report.[2] Undecaprenol was isolated from plant leaves.[3] Solanesol was purchased from 3B Pharmachem International (Wuhan) Co., Ltd. The heptaprenyl-, hexaprenyl-, and tetraprenyl-phosphate were synthesized as described in our previous report.[1,4]

Chemistry

Compound 7. Compound **7** was carried out from **2** as described for the preparation of **5** (33mg, 46%). ^{1}H NMR (600 MHz, D_2O): δ 7.95 (d, 1H, J = 8.4 Hz), 5.95–5.98 (m, 2H), 5.45 (dd, 1H, J = 3.6 and 7.2 Hz), 4.32–4.36 (m, 2H), 4.27–4.30 (m, 2H), 4.10–4.24 (m, 6H), 3.92–3.95 (m, 1H), 3.74–3.86 (m, 3H), 3.63 (dd, 1H, J = 9.0 and 10.2 Hz), 2.97–3.00 (m, 2H), 2.26–2.30 (m, 2H), 2.01–2.13 (m, 1H), 2.00 (s, 3H), 1.89–1.95 (m, 1H), 1.78–1.84 (m, 1H), 1.65–1.71 (m, 3H), 1.36–1.42 (m, 8H); ^{13}C NMR (150 MHz, D_2O): δ 179.0, 177.7, 175.7, 174.7, 174.1, 173.9, 166.1, 151.7, 141.6, 102.6, 94.6, 88.4, 83.1, 79.8, 77.9, 73.7, 72.8, 72.2, 69.6, 67.9, 64.9, 60.2, 54.7, 54.4, 53.3, 49.5, 39.2, 32.1, 30.9, 27.9, 26.2, 22.1, 22.0, 18.6. HRMS calcd for $[C_{34}H_{55}N_7O_{24}P_2+Na]^+$ 1030.2666, found 1030.2659.

Compound 8. Compound **8** was carried out from **2** as described for the preparation of **5**. ^{1}H NMR (600 MHz, D_2O): δ 7.95 (d, 1H, J = 7.8 Hz), 5.96–5.98 (m, 2H), 5.46 (dd, 1H, J = 3.6 and 7.2 Hz), 4.35–4.38 (m, 2H), 4.27–4.31 (m, 2H), 4.16–4.25 (m, 4H), 4.12 (dt, 1H, J = 2.4 and 10.2 Hz), 3.93–3.96 (m, 1H), 3.82–3.88 (m, 2H), 3.78 (t, 1H, J = 9.6 Hz), 3.64 (t, 1H, J = 9.6 Hz), 2.27–2.30 (m, 2H), 2.10–2.13 (m, 1H), 2.00 (s, 3H), 1.87–1.91 (m, 1H), 1.43 (d, 3H, J = 7.2 Hz), 1.40 (d, 3H, J = 6.6 Hz); ^{13}C NMR (150 MHz, D_2O): δ 179.4, 177.8, 175.7, 174.1, 174.0, 166.2, 151.7, 141.6, 102.6, 94.5, 88.3, 83.2, 79.8, 77.9, 73.7, 72.8, 69.6, 67.9, 64.9, 60.2, 54.3, 53.4, 49.5, 31.8, 27.6, 22.0, 18.6, 16.8; HRMS calcd for $[C_{28}H_{43}N_5O_{23}P_2+H]^+$ 880.1902, found 880.1947.

Compound 9. Compound **9** was carried out from **2** as described for the preparation of **5** (24 mg, 32 µmol, 69%). ^{1}H NMR (600 MHz, D_2O): δ 7.94 (d, 1H, J = 7.8 Hz), 5.96–5.98 (m, 2H), 5.46 (dd, 1H, J = 3.6 and 7.2 Hz), 4.34–4.37 (m, 2H), 4.27–4.28 (m, 1H), 4.16–4.25 (m, 3H), 4.08–4.13 (m, 2H), 3.93–3.96 (m, 1H), 3.81–3.88 (m, 2H), 3.64 (dd, 1H, J = 9.0 and 10.2 Hz), 3.78 (t, 1H, J = 9.6 Hz), 2.00 (s, 3H), 1.38 (d, 3H, J = 6.6 Hz), 1.34 (d, 3H, J = 7.8 Hz); ^{13}C NMR (150 MHz, D_2O): δ 179.6, 174.7, 174.1, 166.2, 151.8, 141.6, 102.6, 94.5, 88.3, 83.1, 79.5, 77.8, 73.7, 72.8, 69.6, 68.0, 64.9, 60.2, 53.3, 50.5, 22.0, 18.3, 17.4. HRMS calcd for $[C_{23}H_{36}N_4O_{20}P_2-H]^-$ 749.1319, found 749.1357.

Compound 11. Compound **11** was carried out as described for the preparation of **6**. HRMS calcd for $[C_{52}H_{77}N_{13}O_{30}P_2+H]^+$ 1426.4447, found 1426.4508. The purity of **11** is higher than 95% by analytical anion-exchange HPLC (**Supplementary Figure 7**).

Compound 12. Compound **12** was carried out as described in reference 2. HRMS calcd for $[C_{52}H_{77}N_{13}O_{31}P_2-2H]^-$ 719.7078, found 719.7123.

Compound 13. Compound **13** was carried out as described in reference 2. HRMS calcd for $[C_{52}H_{78}N_{14}O_{30}P_2-2H]^-$ 719.2158, found 719.2195.

Compound 14. Compound **14** was carried out as described for the preparation of **6**. HRMS calcd for $[C_{51}H_{75}N_{13}O_{30}P_2-2H]^{2-}$ 704.7036, found 704.7019. The purity of **14** is higher than 95% by analytical anion-exchange HPLC (**Supplementary Figure 7**).

Compound 15. Compound **15** was carried out as described for the preparation of **6**. HRMS calcd for $[C_{51}H_{75}N_{13}O_{30}P_2-2H]^{2-}$ 704.7036, found 704.7041. The purity of **15** is higher than 95% by analytical anion-exchange HPLC (**Supplementary Figure 7**).

Compound 16. Compound **16** was carried out as described for the preparation of **6**. HRMS calcd for $[C_{51}H_{77}N_{13}O_{28}P_2-H]^-$ 1380.4392, found 1380.4335. The purity of **16** is higher than 95% by analytical anion-exchange HPLC (**Supplementary Figure 7**).

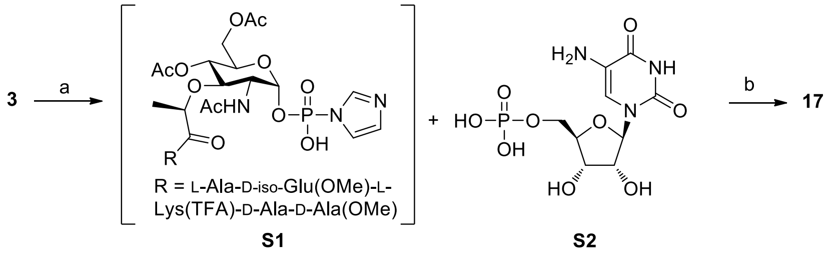

Synthesis of compound 17. Reagents and conditions: (a) CDI, DMF/THF, RT, 2 h, then MeOH; (b) i. 1*H*-tetrazole, DMF/THF, RT, 24 h, ii. LiOH, MeOH, 0 °C, 4 h, iii. NBD-X-OSu, $NaHCO_3$, H_2O, DMF, RT, 2 h, 31% over four steps.

Compound 17. A mixture of **3** (34.0 mg, 30 μmol) and 1,1'-carbonyldiimidazole (10.5 mg, 60 μmol) in DMF/THF (1 mL, v/v = 1:1) was stirred at RT. After stirring for 2 h, anhydrous MeOH (0.2 mL) was added to the reaction for quenching the excess 1,1'-carbonyldiimidazole. After another 1 h stirring, the reaction mixture was concentrated to give **S1**. Compound **S1** was directly used without further purification. To a solution of **S1** in DMF/THF (1mL, v/v = 1:1), 1*H*-tetrazole (3.0 mg, 40 μmol) and 5'-NH_2-UMP (16.0 mg, 40 μmol) were added, and the reaction mixture was stirred at RT for 24 h. After the starting materials were consumed, the residue was concentrated and purified by cc ($CHCl_3$/MeOH/H_2O = 60:25:4, silica gel) to give the pyrophosphate intermediate. This intermediate was dissolved in 0.5 M LiOH in MeOH/H_2O (5 mL, v/v = 1:1) and stirred at 0 °C for 4 h, for the global deprotection. The reaction was then neutralized by 1.0 N $HCl_{(aq)}$, concentrated, and purified by a cc (iPrOH/$NH_4OH_{(aq)}$ = 9:1 to 2:1, silica gel) to give amino-Park's nucleotide as white solid. Compound **17** was carried out from amino-Park's nucleotide as described for the preparation of **6** (13.3 mg, 9.3 μmol, 31% over 4 steps). **17**: HRMS calcd for $[C_{52}H_{78}N_{14}O_{30}P_2{-}H]^-$ 1439.4400, found 1439.4389. The purity of **17** is higher than 95% by analytical anion-exchange HPLC (**Supplementary Figure 7**).

Compound 18. Compound **18** was carried out as described for the preparation of **17**. HRMS calcd for $[C_{23}H_{37}N_5O_{20}P_2{+}H]^+$ 766.1585, found 766.1571.

Compound 19. Compound **19** was carried out as described for the preparation of **17**. ^{1}H NMR (600 MHz, D_2O): δ 8.10 (s, 1H), 5.94 (d, 1H, J = 4.8 Hz), 5.46 (dd, 1H, J = 3.0 and 7.2 Hz), 4.38 (dd, 1H, J = 4.8 and 5.4 Hz), 4.34 (dd, 1H, J = 4.8 and 5.4 Hz), 4.15–4.28 (m, 5H), 4.03–4.11 (m, 4H), 3.61–3.94 (m, 8H), 2.16 (s, 3H), 2.00, (s, 3H), 1.35–1.39 (m, 6H); ^{13}C NMR (150 MHz, D_2O): δ 179.4, 174.7 (×2), 174.5, 174.1, 162.1, 150.7, 136.7, 112.7, 94.5, 89.0, 83.0, 79.5, 77.8, 73.8, 72.9, 69.5, 69.3, 68.1, 60.3, 50.4, 22.0, 21.9, 18.3, 17.3. HRMS calcd for $[C_{25}H_{39}N_5O_{21}P_2{+}H]^+$ 808.1691, found 808.1611.

Compound 20. Compound **20** was carried out as described for the preparation of **17**. ^{1}H NMR (600 MHz, D_2O): δ 8.12 (s, 1H), 7.24–7.30 (m, 4H), 5.93 (d, 1H, J = 4.8 Hz), 5.54 (dd, 1H, J = 3.0 and 7.2 Hz), 4.38 (dd, 1H, J = 4.8 and 5.4 Hz), 4.35 (dd, 1H, J =

4.8 and 5.4 Hz), 4.15–4.27 (m, 5H), 4.03–4.11 (m, 1H), 3.94–3.95 (m, 1H), 3.75–3.87 (m, 4H), 3.61–3.65 (m, 2H), 2.33 (s, 3H), 1.99, (s, 3H), 1.36–1.40 (m, 6H); ^{13}C NMR (150 MHz, D_2O): δ 178.4, 175.0 (×2), 174.2, 174.1, 161.8, 150.7, 137.4, 136.4, 131.4, 129.4, 129.3, 112.7, 94.5, 89.1, 83.0, 79.5, 77.8, 73.5, 72.9, 72.8, 69.4, 68.1, 64.9, 60.2, 53.2, 49.8, 41.5, 22.0, 20.1, 18.3, 16.9. HRMS calcd for $[C_{32}H_{45}N_5O_{21}P_2{-}H]^-$ 896.1999, found 896.2061.

Compound 21. Compound **21** was carried out as described for the preparation of **17**. ^{1}H NMR (600 MHz, D_2O): δ 8.15 (s, 1H), 7.25–7.30 (m, 4H), 5.93 (d, 1H, J = 4.8 Hz), 5.54 (dd, 1H, J = 3.0 and 7.2 Hz), 4.37–4.40 (m, 1H), 4.35 (dd, 1H, J = 4.8 and 5.4 Hz), 4.12–4.27 (m, 6H), 4.07 (q, 1H, J = 6.6 Hz), 3.85 (dd, 1H, J = 3.0 and 10.8 Hz), 3.69–3.78 (m, 4H), 3.59–3.65 (m, 1H), 2.31 (s, 3H), 2.02, (s, 3H), 1.35–1.41 (m, 6H); ^{13}C NMR (150 MHz, D_2O): δ 179.2, 174.9, 174.5, 174.4, 174.3, 161.8, 150.8, 137.4, 136.4, 131.4, 129.4, 129.3, 112.7, 94.7, 89.1, 83.0, 76.8, 76.0, 73.6, 71.7, 69.5, 69.3, 67.6, 64.9, 60.9, 50.3, 48.9, 41.5, 22.1, 20.1, 18.7, 17.3. HRMS calcd for $[C_{32}H_{45}N_5O_{21}P_2{-}H]^-$ 896.1999, found 896.2017.

Compound 22. Compound **22** was carried out as described for the preparation of **17**. ^{1}H NMR (600 MHz, D_2O): δ 8.14 (s, 1H), 7.31 (d, 2H, J = 7.2 Hz), 7.27 (d, 2H, J = 7.2 Hz), 5.95 (d, 1H, J = 4.8 Hz), 5.49 (d, 1H, J = 5.4 Hz), 4.13–4.40 (m, 10 H), 3.91–3.97 (m, 1H), 3.65–3.87 (m, 11 H), 3.00 (t, 1H, J = 7.8 Hz), 2.35 (s, 3H), 2.31 (t, 1H, J = 7.8 Hz), 2.16–2.19 (m, 1H), 1.98−2.00 (m, 4H), 1.65–1.90 (m, 5H), 1.31–1.44 (m, 12H). HRMS calcd for $[C_{49}H_{74}N_{10}O_{27}P_2{-}H]^-$ 1295.4116, found 1295.4071.

Biology

Site–directed mutagenesis of MraY. Site-directed mutagenesis of the *B. subtilis* MBP-tagged MraY enzyme was performed directly on the pMAL-BS-MraY-16 expression plasmid by using the "Quickchanged XL Site-Directed Mutagenesis Kit" from stratagene and the oligonucleotides shown below. Mutations introduced in the *mraY* gene sequence were checked by DNA sequencing.

Mutation	Name	Sequence
T51A	MraY-T51A-F	5'-CAGAAAAAATCAGGGGCACCGACAATGGGCGGGGTC-3'
	MraY-T51A-R	5'-GACCCCGCCCATTGTCGGTGCCCCTGATTTTTTCTG-3'
T53A	MraY-T53A-F	5'-AAATCAGGGACACCGGCAATGGGCGGGGTCATGATC-3'
	MraY-T53A-R	5'-GATCATGACCCCGCCCATTGCCGGTGTCCCTGATTT-3'
K102A	MraY-K102A-F	5'-GGCTTTTTAGATGATTACATCGCGGTTGTCATGAAGCGCAATCTTGG-3'
	MraY-K102A-R	5'-CCAAGATTGCGCTTCATGACAACCGCGATGTAATCATCTAAAAAGCC-3'
K226A	Mray-K226A-F	5'-GTATTTAATGCTCATCCGGCCGCAGTTTTTATGGGAGATACGGG-3'
	Mray-K226A-R	5'-CCCGTATCTCCCATA AAA ACTGCGGCCGGATGAGCATTAAATAC-3'
F228A	MraY-F228A-F	5'-GCTCATCCGGCCAAAGTTGCTATGGGAGATACGGGATCG-3'
	MraY-F228A-R	5'-CGATCCCGTATCTCCCATAGCAACTTTGGCCGGATGAGC-3'
Q271A	Mray-Q271A-F	5'-GAGACATTATCCGTTATTTTGGCGGTCATCAGCTTTAAAACGACAGG-3'
	Mray-Q271A-R	5'-CCTGTCGTTTTAAAGCTGATGACCGCCAAAATAACGGATAATGTCTC-3'
R281A	MraY-R281A-F	5'-CAGCTTTAAAACGACAGGTAAAGCAATCTTTAAAATGAGTCCGCTTC-3'
	MraY-R281A-R	5'-GAAGCGGACTCATTTTAAAGATTGCTTTACCTGTCGTTTTAAAGCTG-3'
W297A	MraY-W297A-F	5'-CACCATTATGAGCTTGTCGGCGCGTCTGAATGGAGAGTAGTCG-3'
	MraY-W297A-R	5'-CGACTACTCTCCATTCAGACGCGCCGACAAGCTCATAATGGTG-3'

Determine kinetic parameters by MraY fluorescence enhancement assay. The fluorescence enhancement assay was performed in 384-well plate as described by Bugg et al. with slight modifications.[5] The reaction mixture containing $MraY_{BS}$ (10 μg/mL), $C_{55}P$ (200 μM) in 30 μL reaction buffer (30 mM Tris, 10 mM $MgCl_2$, 10 mM NaCl, 0.1 mM Tween-20, 2.5% DMSO, pH 8.0) were pre-incubated at 37 °C for 15 min. The reactions were initiated by the addition of various concentrations of NBD-Park's nucleotide **6** (0 to 160 μM). For inhibition study, a various concentrations of inhibitors were added before the addition of substrate **6**. The continuous fluorescence increase over 60 min was monitored with λ_{ex} 466 nm/λ_{em} 535 nm by fluorescence microplate reader (SpectraMax M5, Molecular Devices, LLC) at 37 °C. Linear regressions of increased fluorescent units (FLU) versus time (min) were used to calculate the initial velocities for each reaction. The kinetic parameters values (K_i, K_M, and V_{max}) were obtained by nonlinear regression analysis using the Lineweaver-Burk equation with the GraphPad Prism program (GraphPad Software, San

Diego, CA).

Construction of molecular modelings of MraY with Park's nucleotides. The molecular modeling work was conducted by using Schrödinger Suites (Schrödinger Inc, USA). Initially, a crystal structure of a MraY was downloaded from the RCSB Protein Data Bank (PDB coded 4J72) and used as a template to build the MraY molecule of Bacillus subtilis. The molecular structure was then refinement by using the Protein Preparation Wizard. Briefly, all essential hydrogen atoms were added to the structure model followed by assigning charges and solvation parameters before energy minimization. The molecular complexes of MraY with Park's nucleotides were generated by computational docking method enabled by Glide (Schrödinger Inc, USA) with OPLS2005/AA force field.[6] Additionally, metal ions of Mg and Ni were considered in the modeling, and the starting position of C55P was manually selected according to the report.[7] The molecular structures of Park's nucleotides were prepared and energy minimized using LigPrep with a consideration of tautomer in calculated pKa. For a final interacting complex, the Gilde XP mode was applied, allowing flexible conformational search of the compounds against the target. With detail analyzing the obtained results of mutagenesis and substrate activity, one ligand-enzyme complex model was elected from the five top scored docking poses by visual examination.

Determination of Minimal Inhibition Concentration (MIC). *B. subtilis* (BCRC 10614) and *S. aureus* (BCRC 11863) was obtained from Bioresource Collection and Research Center (BCRC), Taiwan. The minimal inhibitory concentration of tested compounds was determined following the NCCLS standard. The experiments were conducted in 96-well microtiter plates using 16-point 2-fold dilutions in Muller-Hilton broth. Exponentially growing cells at 5×10^5 cells/mL were incubated with test compounds at various concentrations in a final volume of 100 μL. After an 18 to 24 h incubation at 37 °C, the minimal concentration of the compound that prevents at least 95% bacterial growth was determined as the MIC value of a given molecule.

Supplementary Tables

Supplementary table 1. Substrate activity study of polyprenol phosphates toward $MraY_{BS}$.

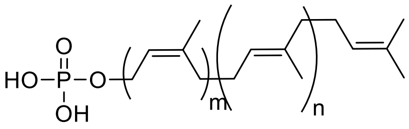

Entry	Lipids	Structure	conversion %[a]
1	Undecaprenol	m = 7; n = 3	96 ± 1
2	Solanesol	m = 0; n = 8	12 ± 2
3	Heptaprenol	m = 6; n = 0	55 ± 3
4	Hexaprenol	m = 5; n = 0	27 ± 6
5	Tetraprenol	m = 3; n = 0	16 ± 5

[a] The assay was performed in buffer (30 mM Tris, 10 mM $MgCl_2$, 10 mM NaCl, 0.1 mM Tween-20, 2.5% DMSO, pH 8.0) containing $MraY_{BS}$ (10 μg/mL), NBD-Park's nucleotide (**6**) (10 μM), lipid phosphates (200 μM), at 37 °C for 1 h. The conversion rates (%) were calculated based on the peak consumption of NBD-Park's nucleotide (**6**) in HPLC analysis. Results for each group (n = 3) are reported as average ± standard deviation.

Supplementary Figures

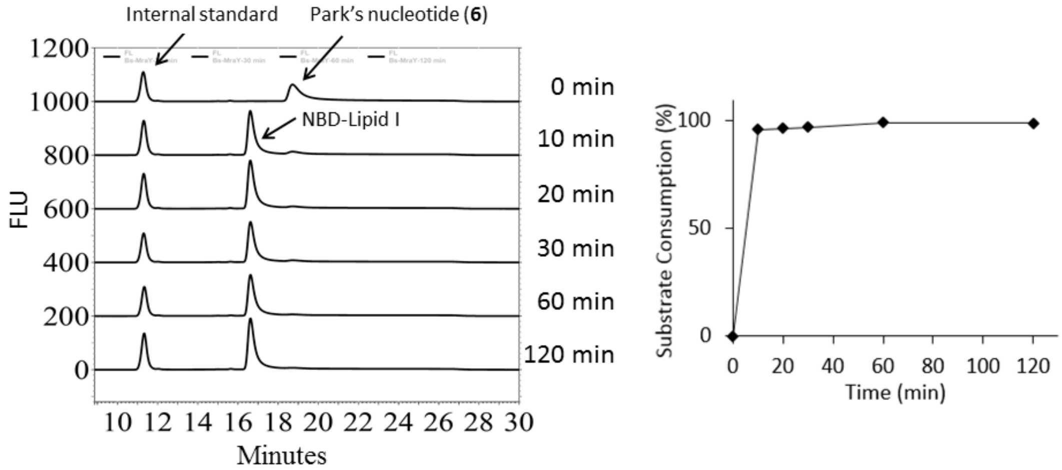

Supplementary figure 1. Substrate utilization of **6** in HPLC-based MraY functional assay.

The assay was performed in buffer (30 mM Tris, 10 mM $MgCl_2$, 10 mM NaCl, 0.1 mM Tween-20, 2.5% DMSO, pH 8.0) containing $MraY_{BS}$ (10 µg/mL), NBD-Park's nucleotide (**6**) (10 µM), 6-(7-Nitro-2,1,3-benzoxadiazol-4-ylamino) hexanoic acid (5 µM) and $C_{55}P$ (200 µM) at 37 °C. The reaction mixtures were heated to 100 °C to stop the transferring reaction at 0, 10, 20, 30, 60 and 120 min. The reaction samples were analyzed on an anion-exchange column by HPLC with a fluorescent detector (λ_{ex} 466 nm/λ_{em} 535 nm). The resulting product of **6** in reaction mixtures was analyzed by HRMS. NBD-Lipid I (C55-PP-MurNAc-D-*iso*-Glu-L-Lys(NBD)-D-Ala-D-Ala) from **6**, calcd for $[C_{98}H_{154}N_{11}O_{25}P_2–H]^-$ 1947.0577, found 1947.0587. NBD-Park's nucleotide **6** was completely consumed in 60 min.

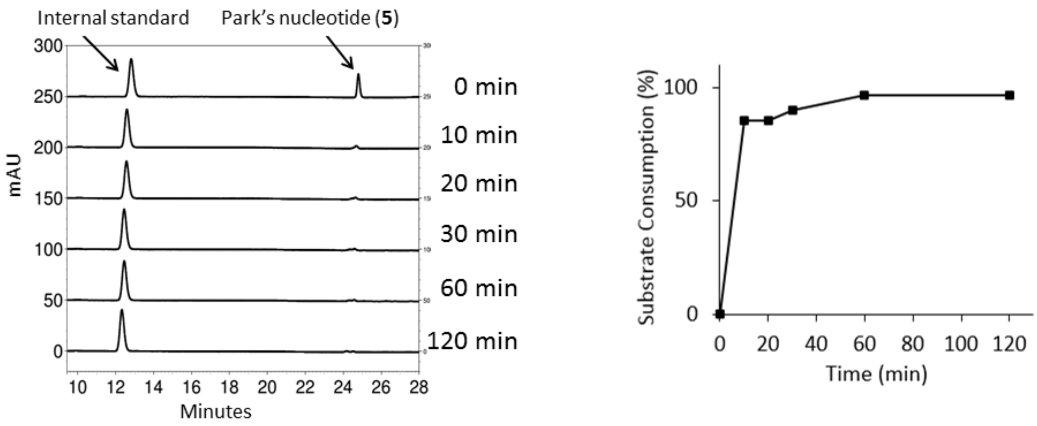

Supplementary figure 2. Substrate utilization of **5** in HPLC-based MraY functional assay.

The reactions were performed with $MraY_{BS}$ (10 μg/mL) by HPLC-based MraY functional assay as described in Methods. The reaction samples were analyzed by RP-C18 HPLC with a UV detector (260 nm) at 0, 10, 20, 30, 60 and 120 min, and uridine (0.01 mg/mL) was added as the internal standard. The resulting products of **5** in reaction mixtures were analyzed by HRMS. Lipid I (C55-PP-MurNAc-D-*iso*-Glu-L-Lys-D-Ala-D-Ala) from **5**, calcd for $[C_{86}H_{143}N_7O_{21}P_2{-}2H]^-$ 834.9828, found 834.9877. Park's nucleotide **5** was completely consumed in 60 min.

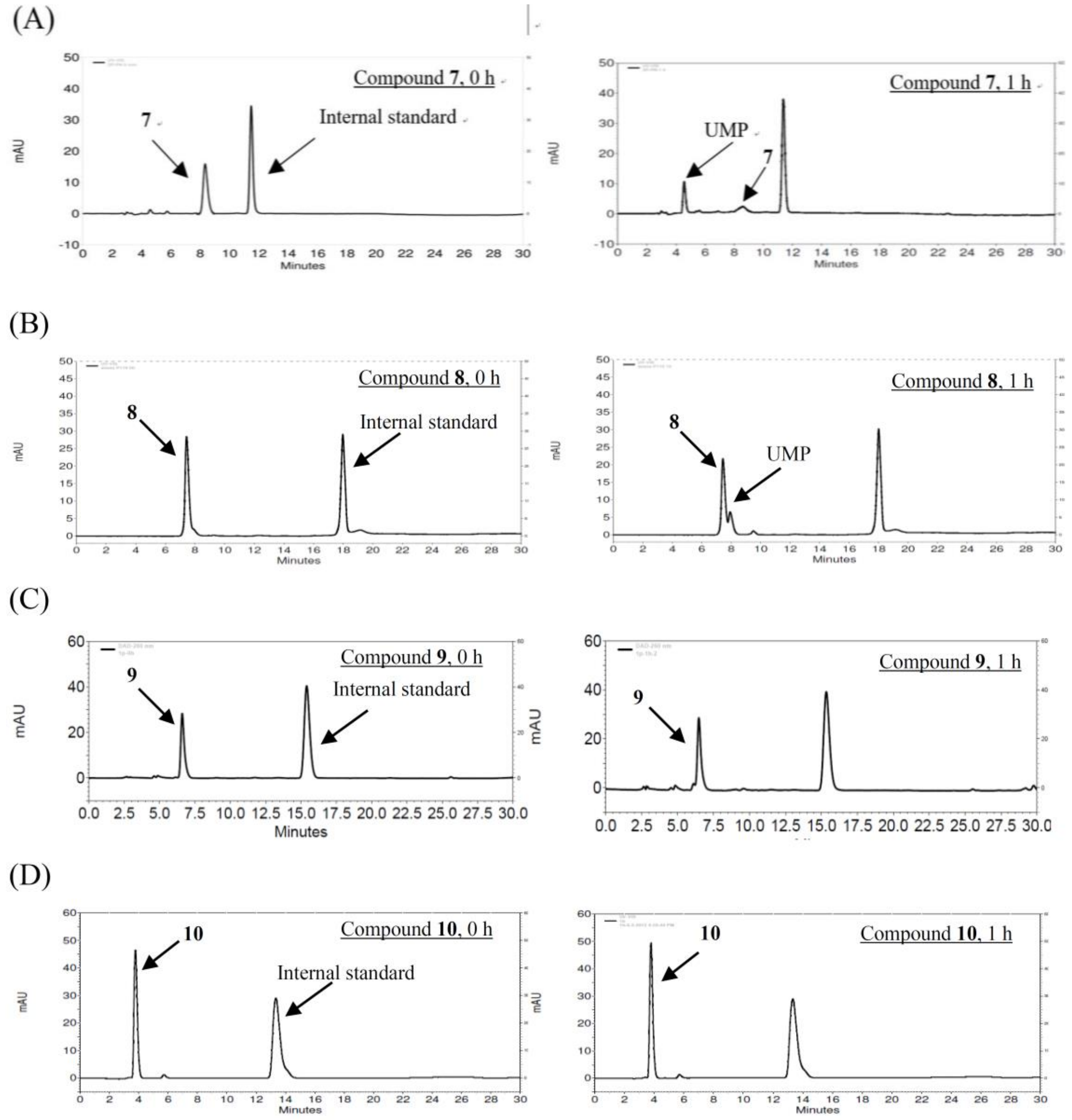

Supplementary figure 3. Substrate utilization of **7-10** in HPLC-based MraY functional assay.

The reactions were performed with $MraY_{BS}$ (10 μg/mL) by HPLC-based MraY functional assay as described in Methods. The reactions of (A) **7**, (B) **8**, (C) **9**, and (D)**10** were analyzed at 0 and 1 h by RP-C18 HPLC with a UV detector (260 nm). The internal standard (uridine 0.01 mg/mL) and the released UMP in figures are indicated. The resulting products of **7** and **8** in reaction mixtures were analyzed by HRMS. Lipid I (C55-PP-MurNAc-D-*iso*-Glu-L-Lys) from **7**, calcd for $[C_{80}H_{133}N_5O_{19}P_2 - 2H]^{-}$ 763.9457, found 763.9481; Lipid I (C55-PP-MurNAc-D-*iso*-Glu) from **8**, calcd for $[C_{74}H_{121}N_3O_{18}P_2 - 2H]^{-}$ 699.8982, found 699.8911.

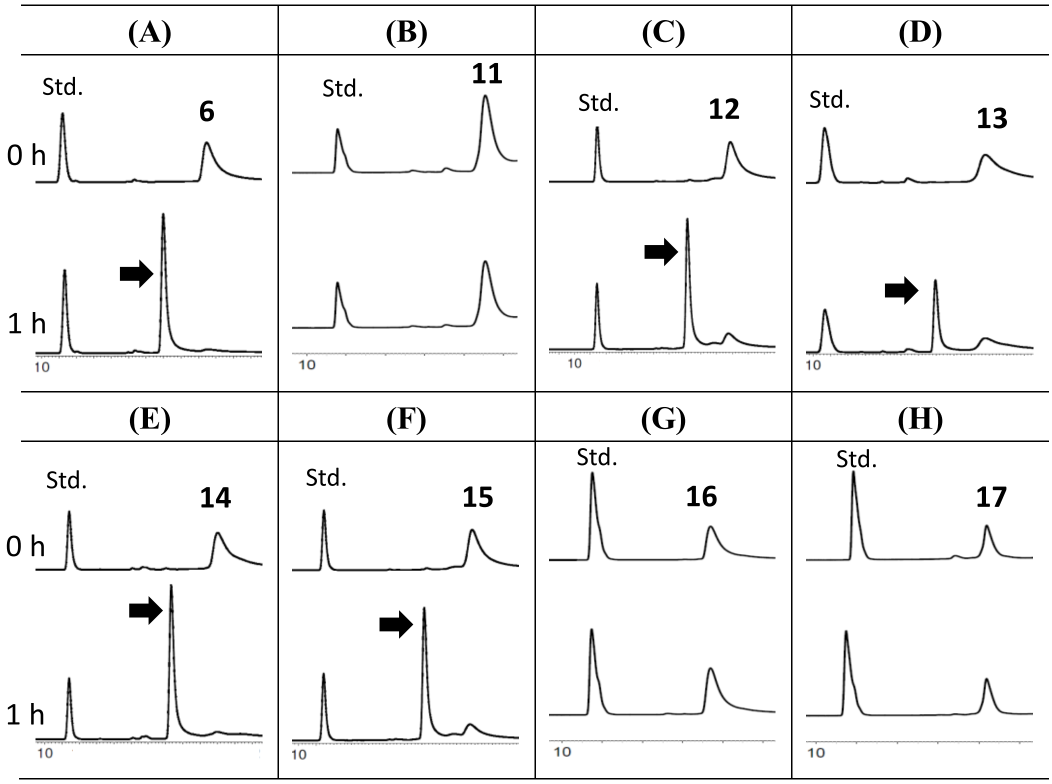

Supplementary figure 4. Substrate utilization of **6** and **11-17** in HPLC-based MraY functional assay.

The reactions were performed with $MraY_{BS}$ (10 μg/mL) by HPLC-based MraY functional assay as described in Methods. The reactions of (A) **6**, (B) **11**, (C) **12**, (D) **13**, (E) **14**, (F) **15**, (G) **16** and (H) **17**, were analyzed at 0 and 1 h on an anion-exchange column by HPLC with a fluorescent detector (λ_{ex} 466 nm/λ_{em} 535 nm). The internal standard (6-[(7-nitro-2,1,3-benzoxadiazol-4-yl)-amino] hexanoic acid) and the corresponding Lipid I products in figures are indicated.

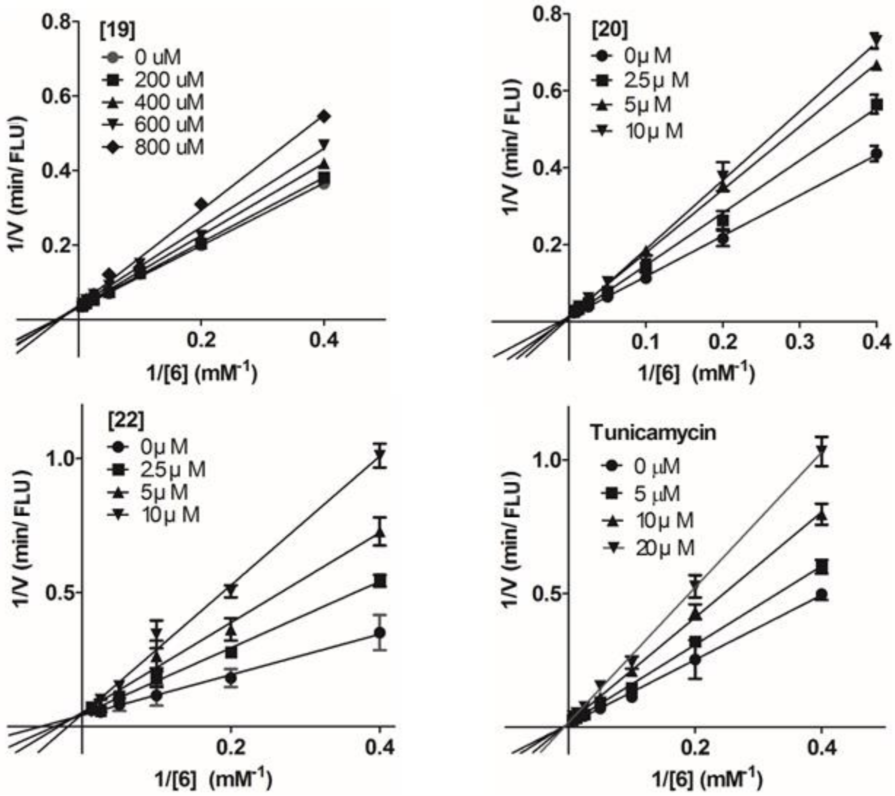

Supplementary figure 5. Competitive inhibition of **19**, **20**, **22** and tunicamycins against $MraY_{BS}$ activity. The inhibitory constant (K_i) of **19**, **20**, **22** and tunicamycins toward MraY was examined by fluorescent enhancement assay as described in Supplementary Methods. All four inhibitors (A) **19**, (B) **20**, (C) **22** and (D) tunicamycins showed competitive patterns in Lineweaver-Burk plot. FLU: fluorescent units. The K_i values of **19**, **20**, **22** and tunicamycins were 764 ±127, 11 ±3, 4 ±1, and 9 ±1 μM, respectively.

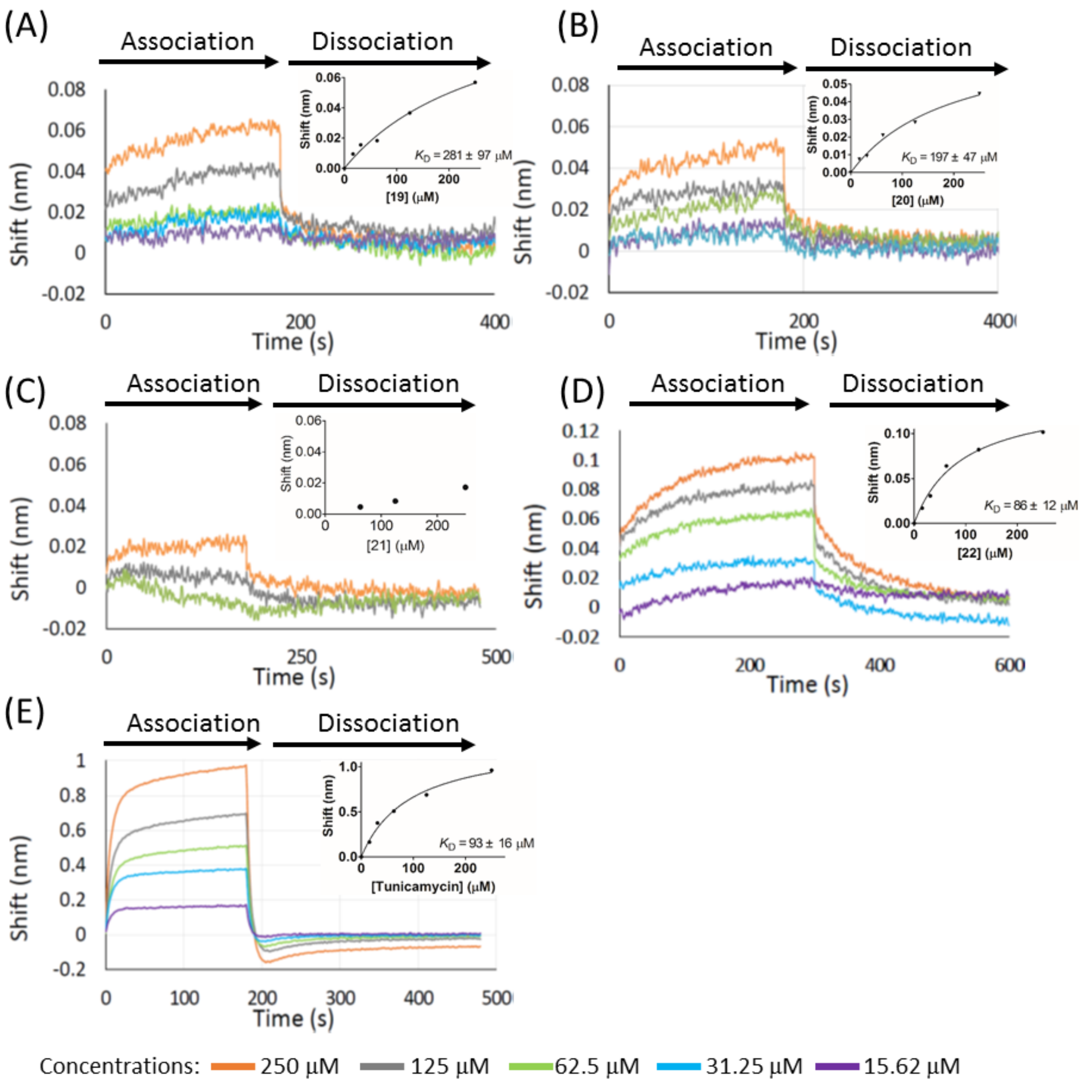

Supplementary figure 6. Evaluation of the binding affinity of Park's analogues (**19-22**) and tunicamycins toward $MraY_{BS}$. The binding affinity was determined by the biolayer interferometry-based binding (BLI) assay as described in Methods. The BLI sensorgrams of (A) **19**, (B) **20**, (C) **21**, (D) **22**, (E) tunicamycins binding to $MraY_{BS}$ are shown. The dissociation constants (K_D) were obtained by nonlinear regression analysis using the specific binding model with the GraphPad Prism program (GraphPad Software, San Diego, CA).

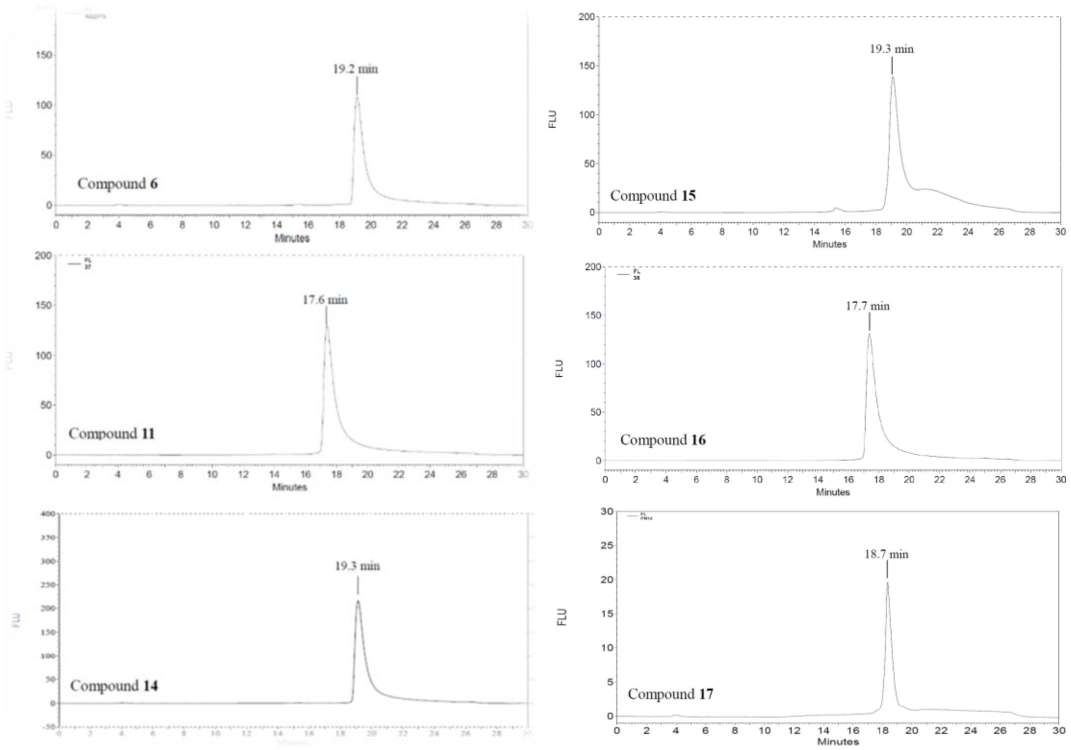

Supplementary figure 7. The purity of NBD-Park's nucleotide analogues **6**, **11**, **14**, **15**, **16** and **17**.

The analysis of NBD-labeled Park's nucleotides was performed on anion exchange column (SAX1, Supelco Co., 5 µm, 4.6 × 250 mm) by HPLC with a linear gradient elution of NH_4OAc (20 mM to 1 M in MeOH) at a flow rate of 1.0 mL/min over 30 min. The fluorescent substrates were monitored with λ_{ex} 466 nm/λ_{em} 535 nm by fluorescence detector. NBD-Park's nucleotide analogues **6**, **11**, **14**, **15**, **16** and **17** showed retention time at 19.2, 17.6, 19.3, 19.3, 17.7 and 18.7 min on fluorescent-HPLC spectra, respectively.

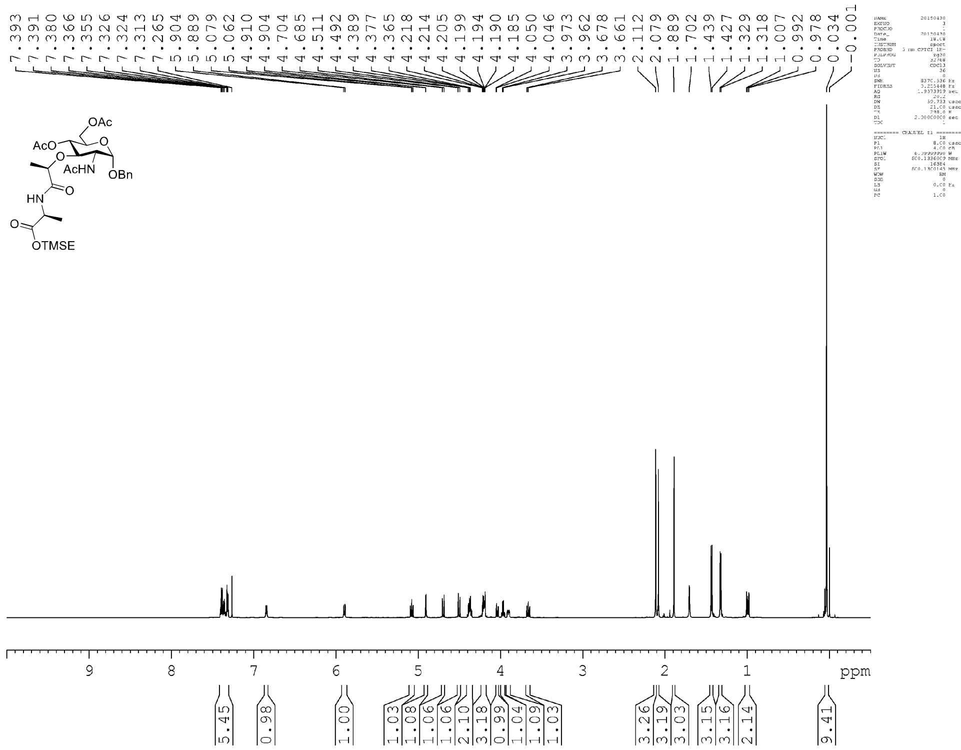

Supplementary figure 8. ^{1}H-NMR Spectra of compound **1** (600 MHz, $CDCl_3$)

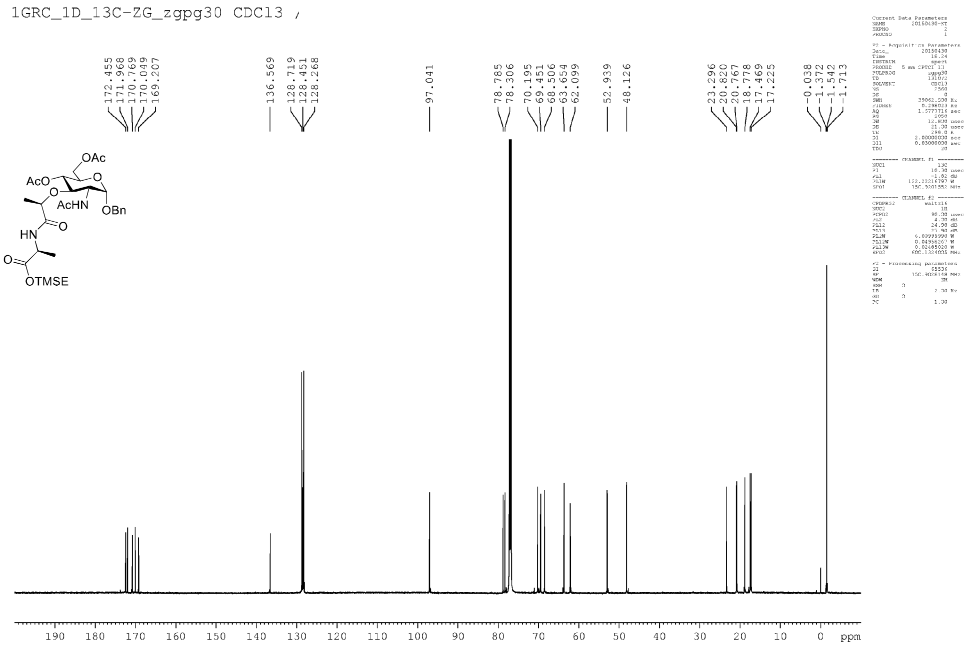

Supplementary figure 9. ^{13}C-NMR Spectra of compound **1** (150 MHz, $CDCl_3$)

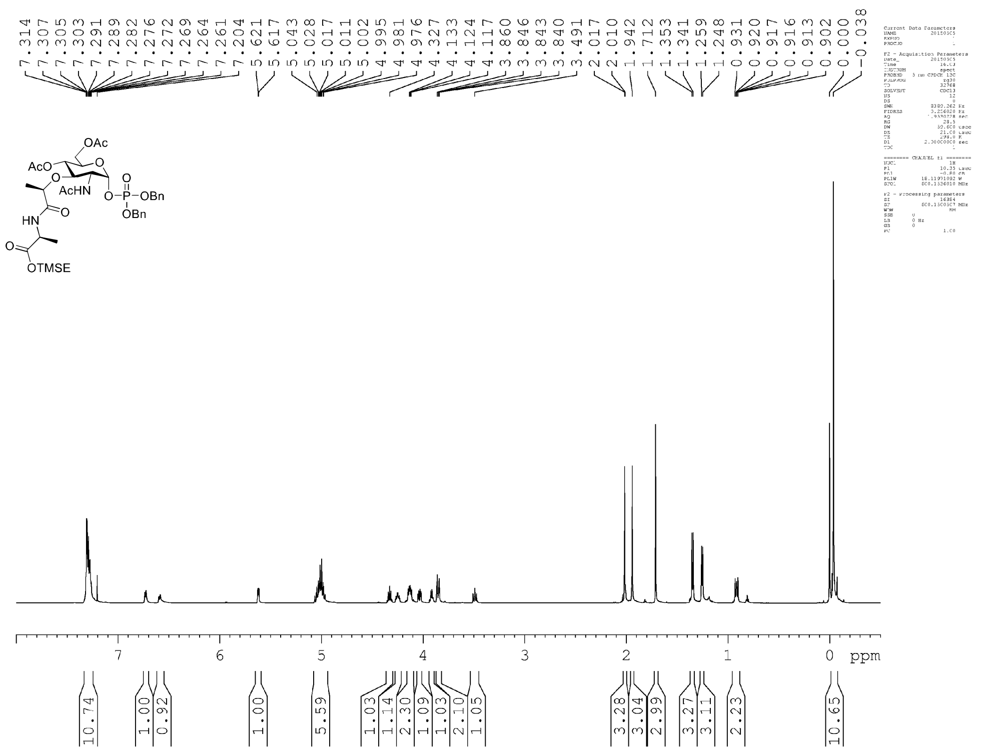

Supplementary figure 10. ^{1}H-NMR Spectra of compound **2** (600 MHz, $CDCl_3$)

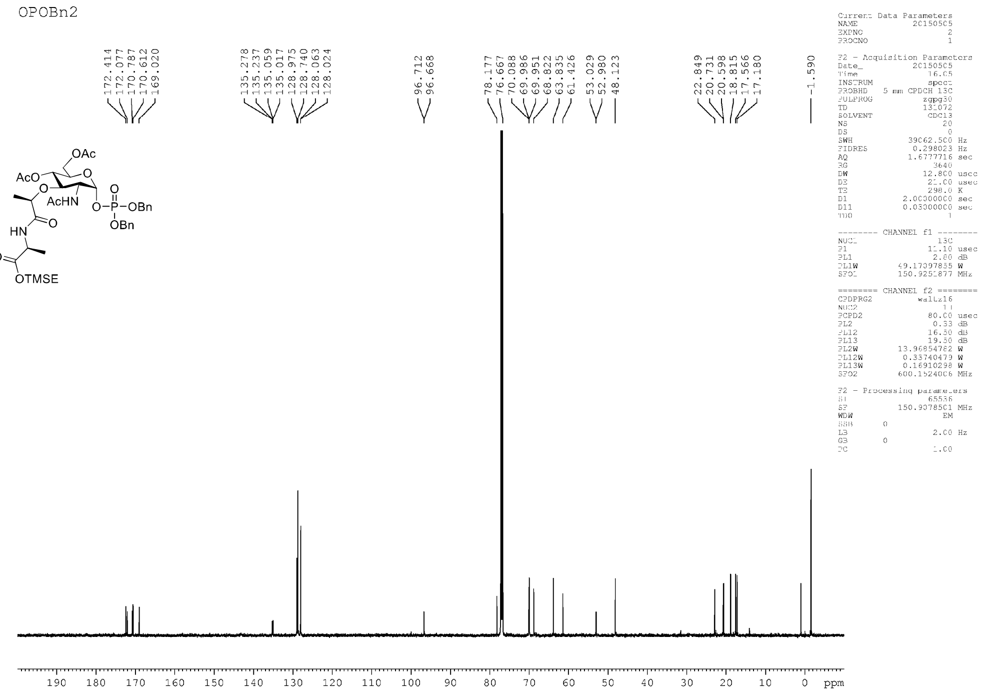

Supplementary figure 11. ^{13}C-NMR Spectra of compound **2** (150 MHz, $CDCl_3$)

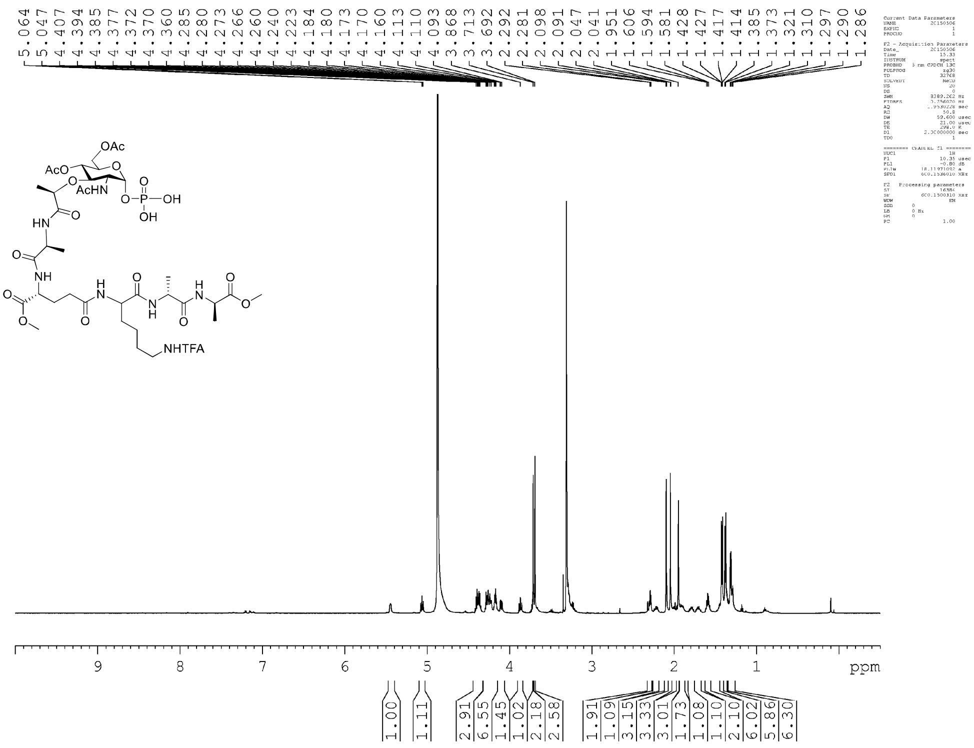

Supplementary figure 12. ^{1}H-NMR Spectra of compound **3** (600 MHz, CD_3OD)

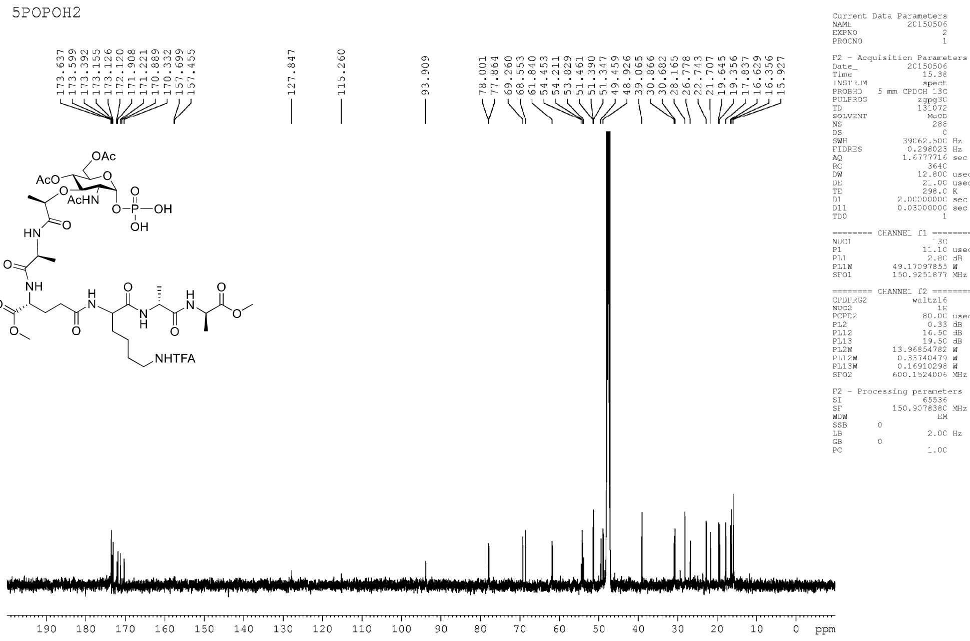

Supplementary figure 13. ^{13}C-NMR Spectra of compound **3** (150 MHz, CD_3OD)

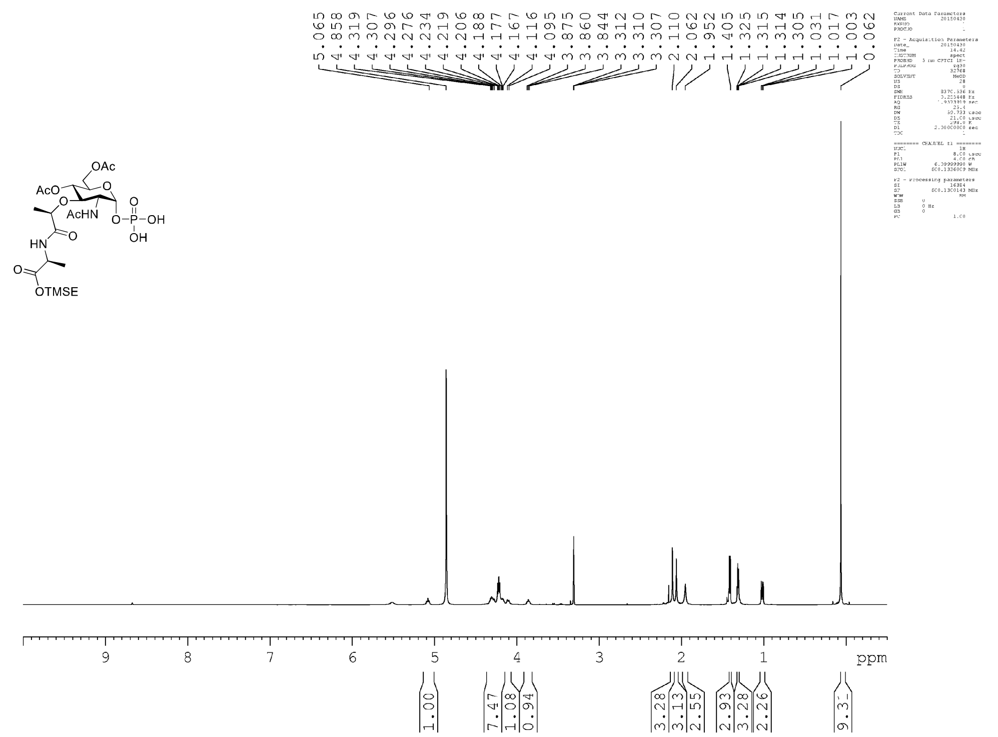

Supplementary figure 14. ^{1}H-NMR Spectra of compound **4** (600 MHz, CD_3OD)

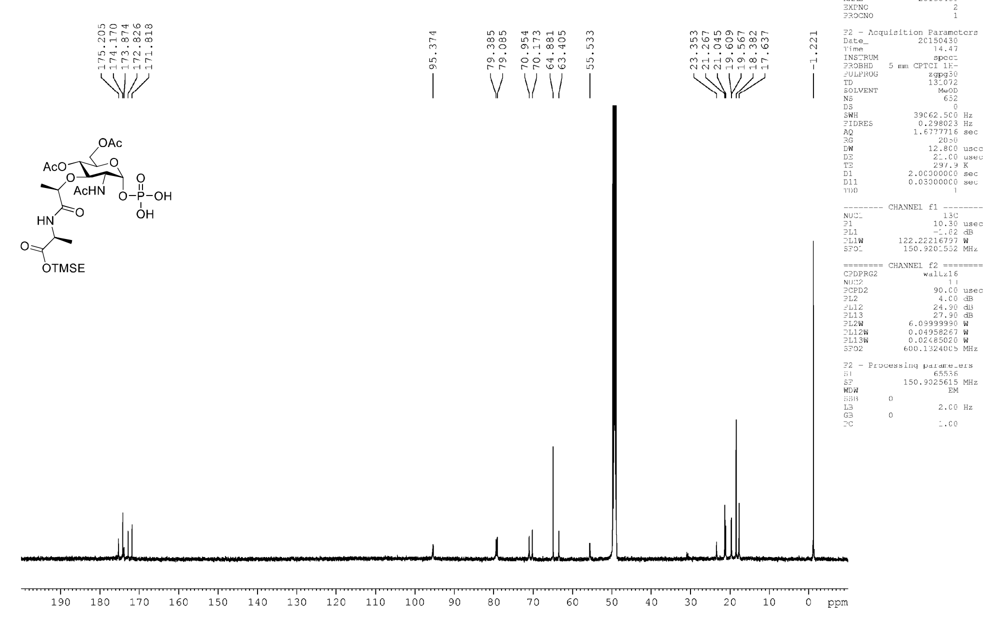

Supplementary figure 15. ^{13}C-NMR Spectra of compound **4** (150 MHz, CD_3OD)

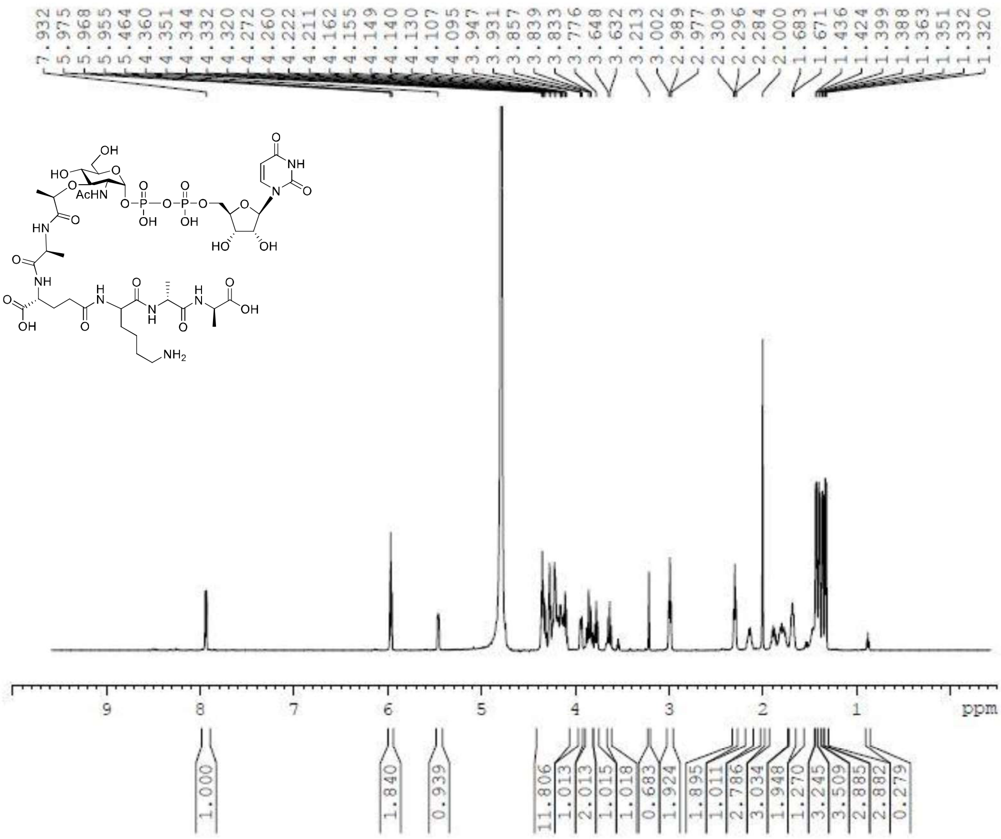

Supplementary figure 16. ^{1}H-NMR Spectra of compound **5** (600 MHz, D_2O)

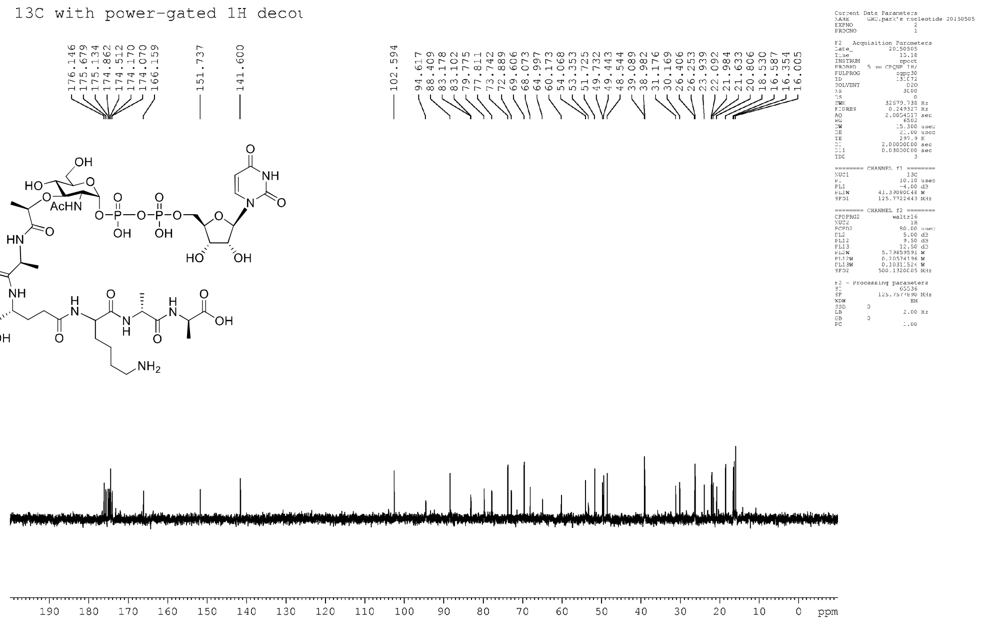

Supplementary figure 17. ^{13}C-NMR Spectra of compound **5** (150 MHz, D_2O)

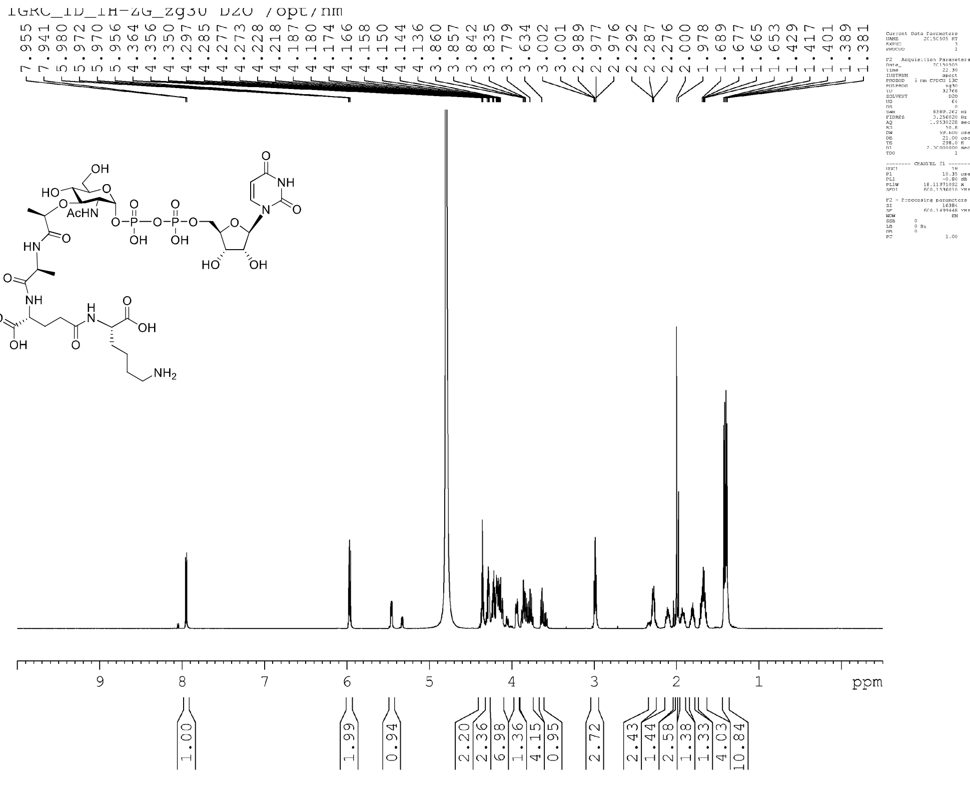

Supplementary figure 18. ^{1}H-NMR Spectra of compound **7** (600 MHz, D_2O)

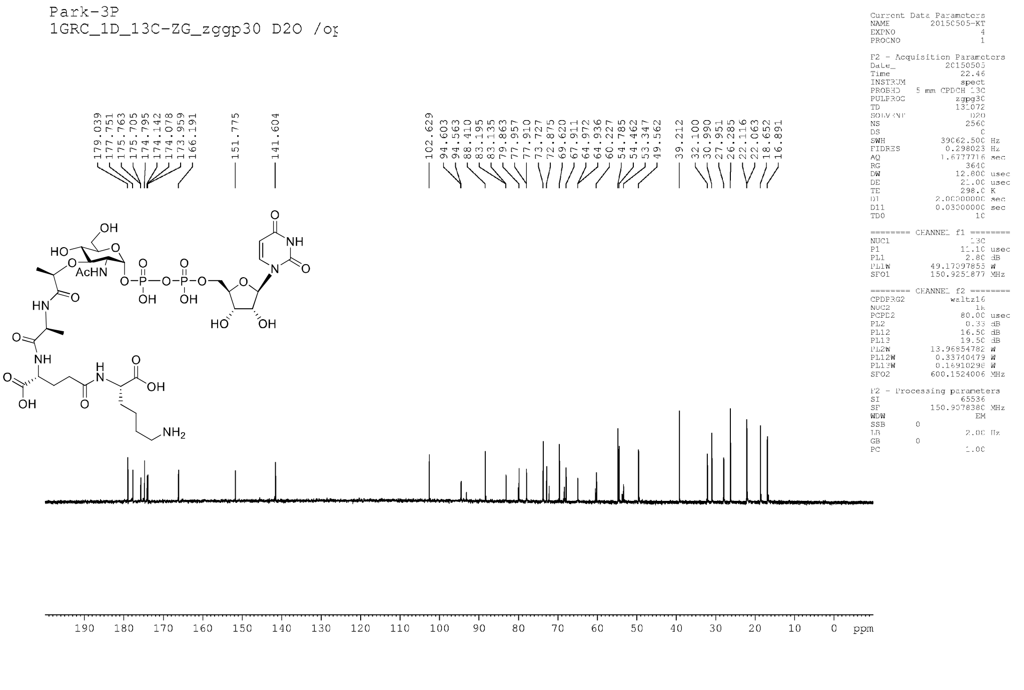

Supplementary figure 19. ^{13}C-NMR Spectra of compound **7** (150 MHz, D_2O)

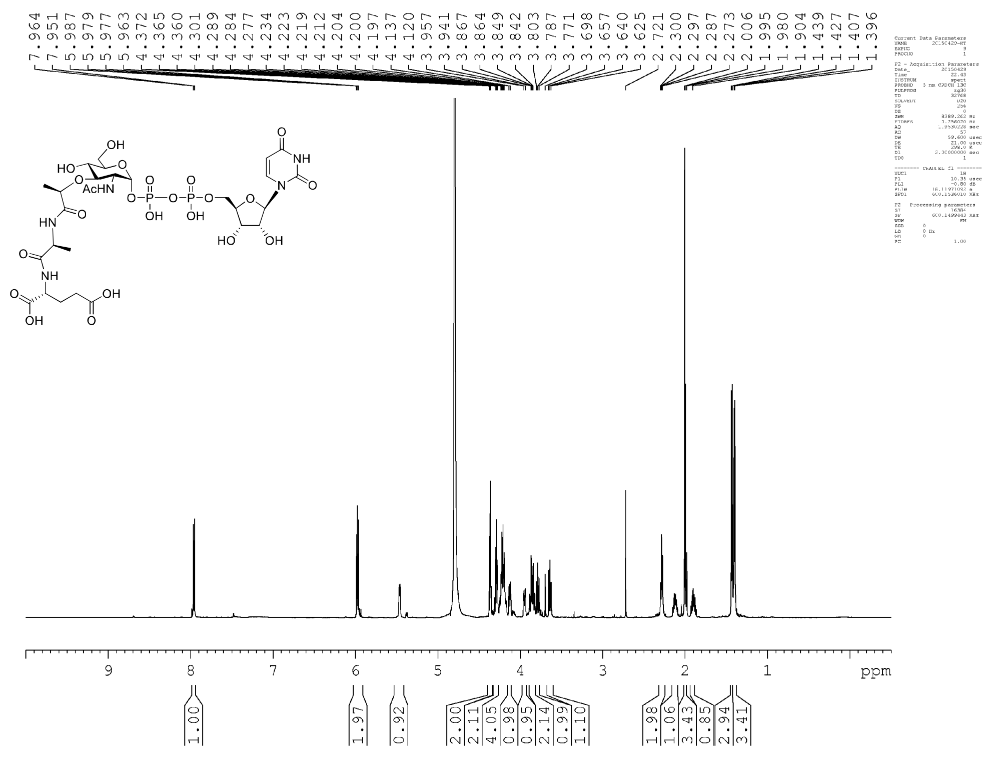

Supplementary figure 20. ^{1}H-NMR Spectra of compound **8** (600 MHz, D_2O)

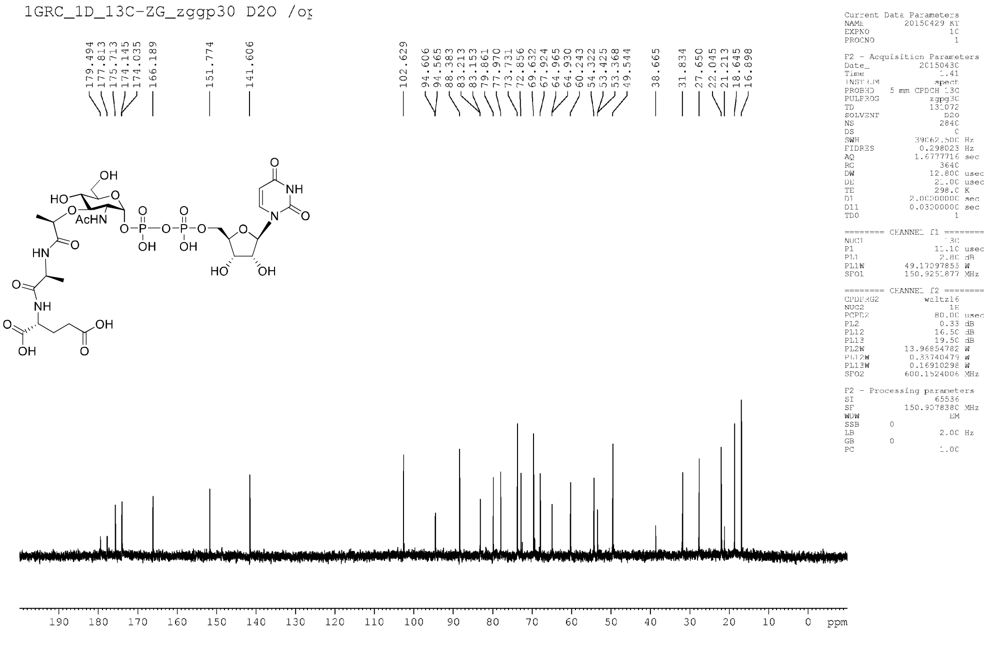

Supplementary figure 21. ^{13}C-NMR Spectra of compound **8** (150 MHz, D_2O)

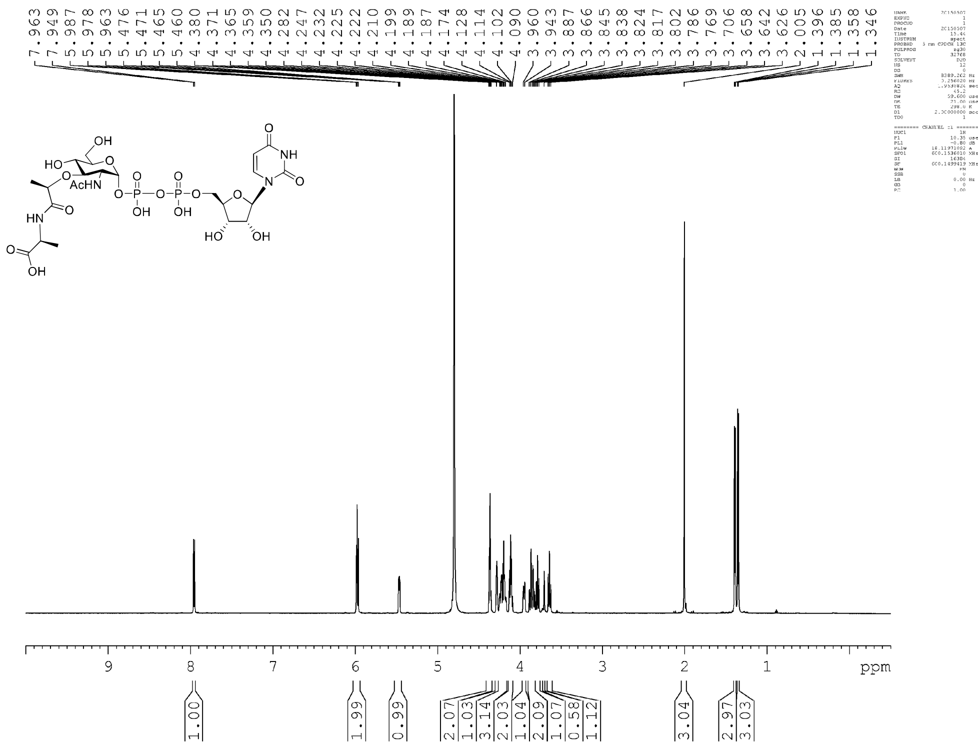

Supplementary figure 22. ^{1}H-NMR Spectra of compound **9** (600 MHz, D_2O)

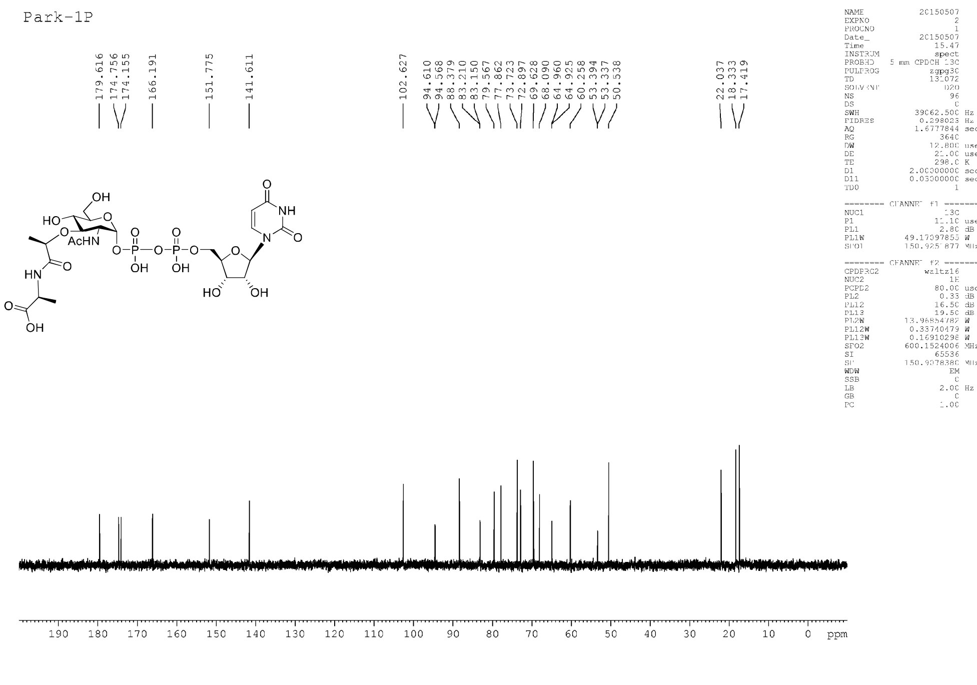

Supplementary figure 23. ^{13}C-NMR Spectra of compound **9** (150 MHz, D_2O)

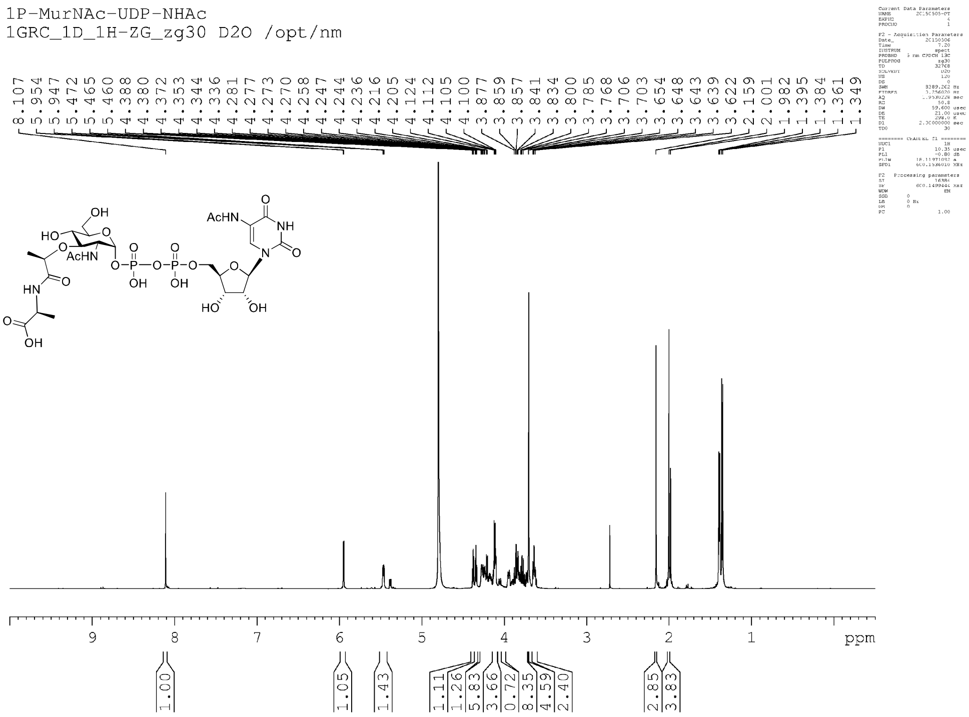

Supplementary figure 24. ^{1}H-NMR Spectra of compound **19** (600 MHz, D_2O)

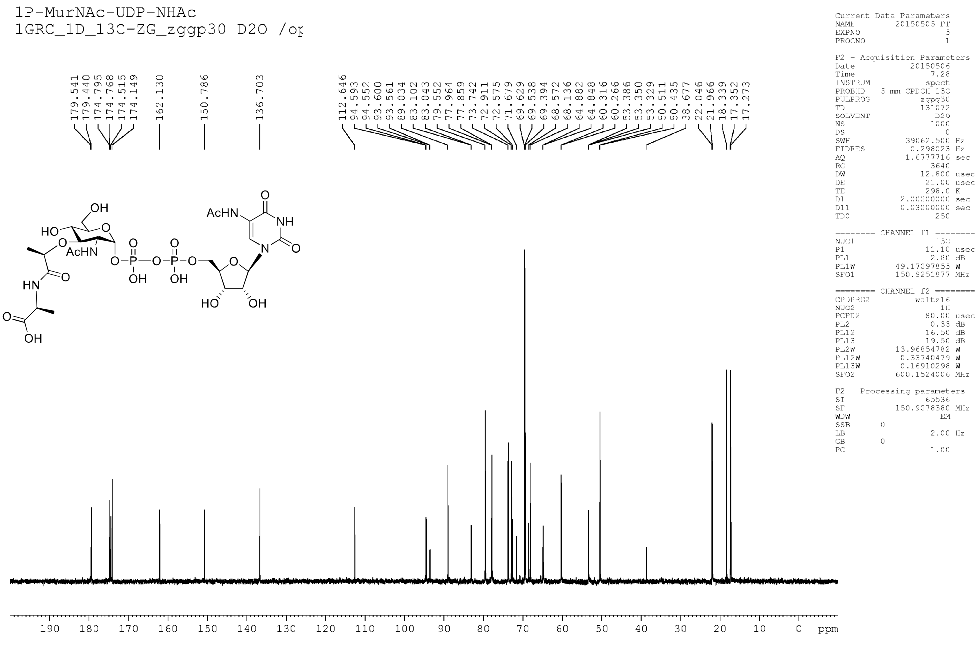

Supplementary figure 25. ^{13}C-NMR Spectra of compound **19** (150 MHz, D_2O)

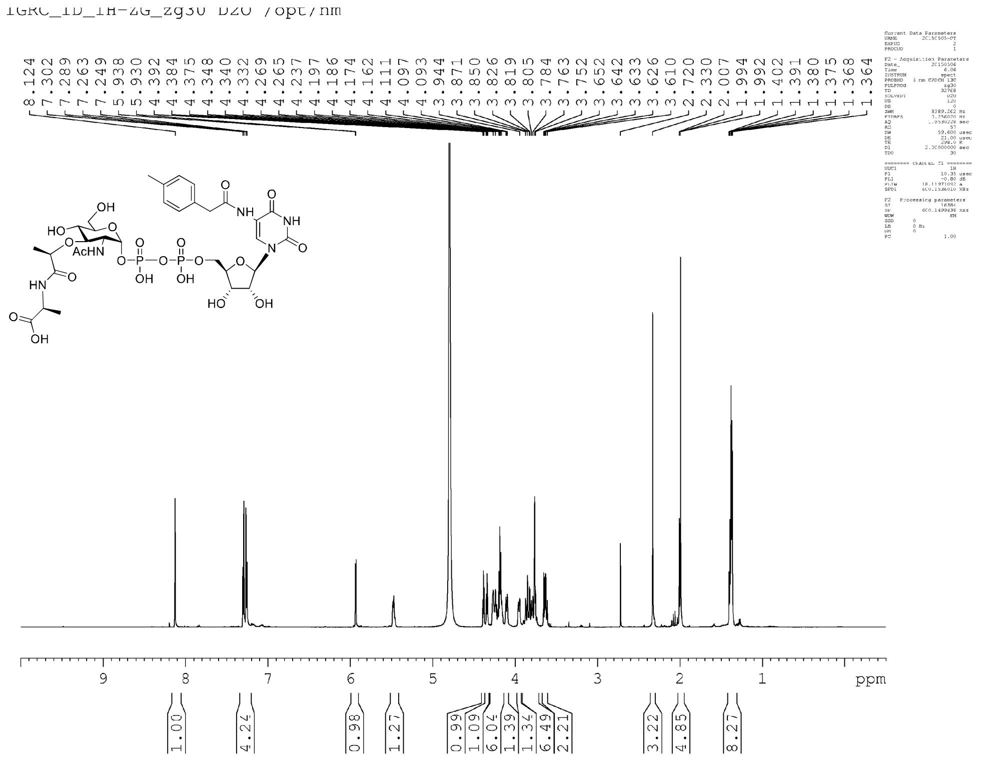

Supplementary figure 26. ^{1}H-NMR Spectra of compound **20** (600 MHz, D_2O)

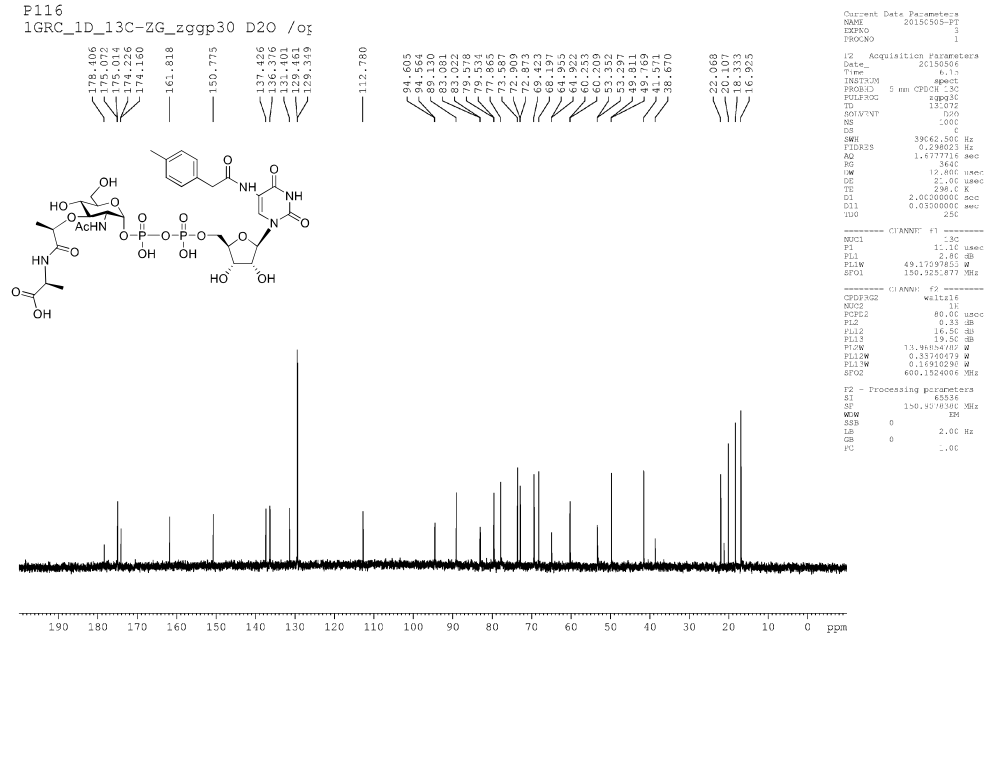

Supplementary figure 27. ^{13}C-NMR Spectra of compound **20** (150 MHz, D_2O)

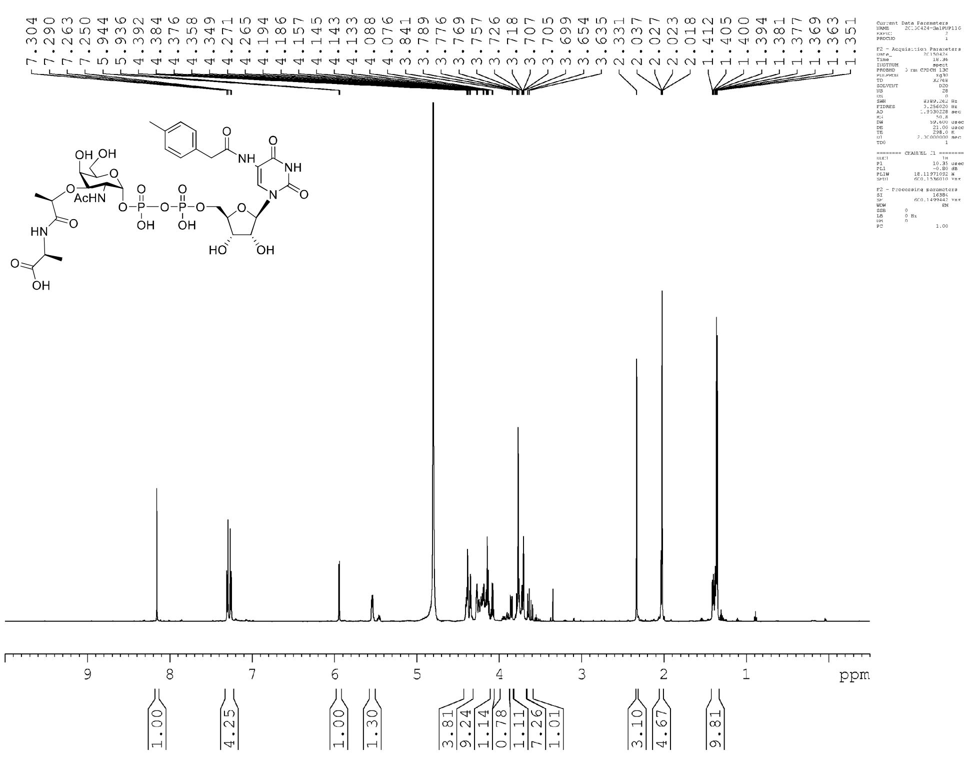

Supplementary figure 28. ^{1}H-NMR Spectra of compound **21** (600 MHz, D_2O)

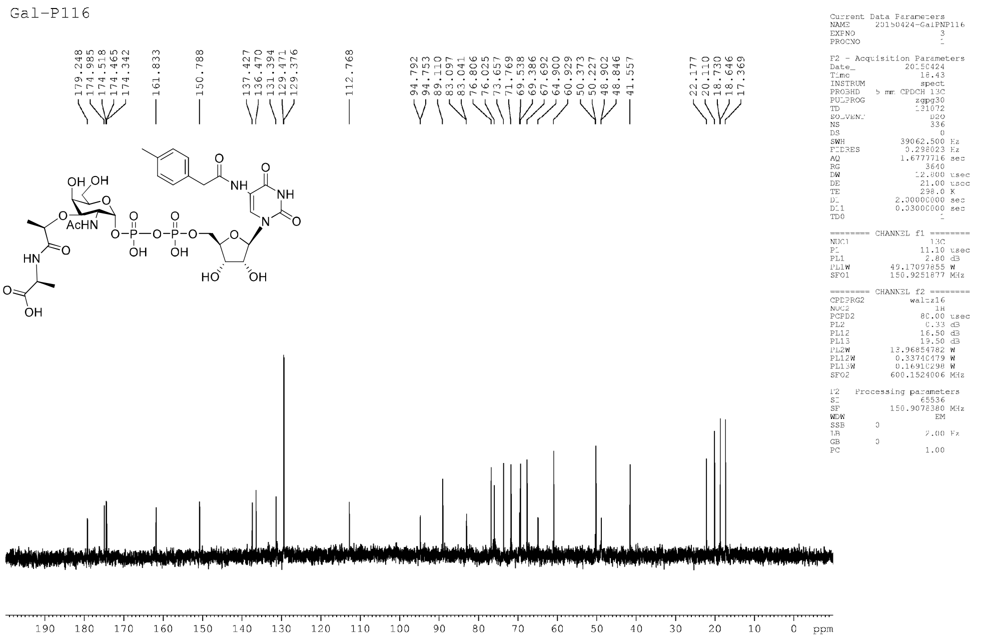

Supplementary figure 29. ^{13}C-NMR Spectra of compound **21** (150 MHz, D_2O)

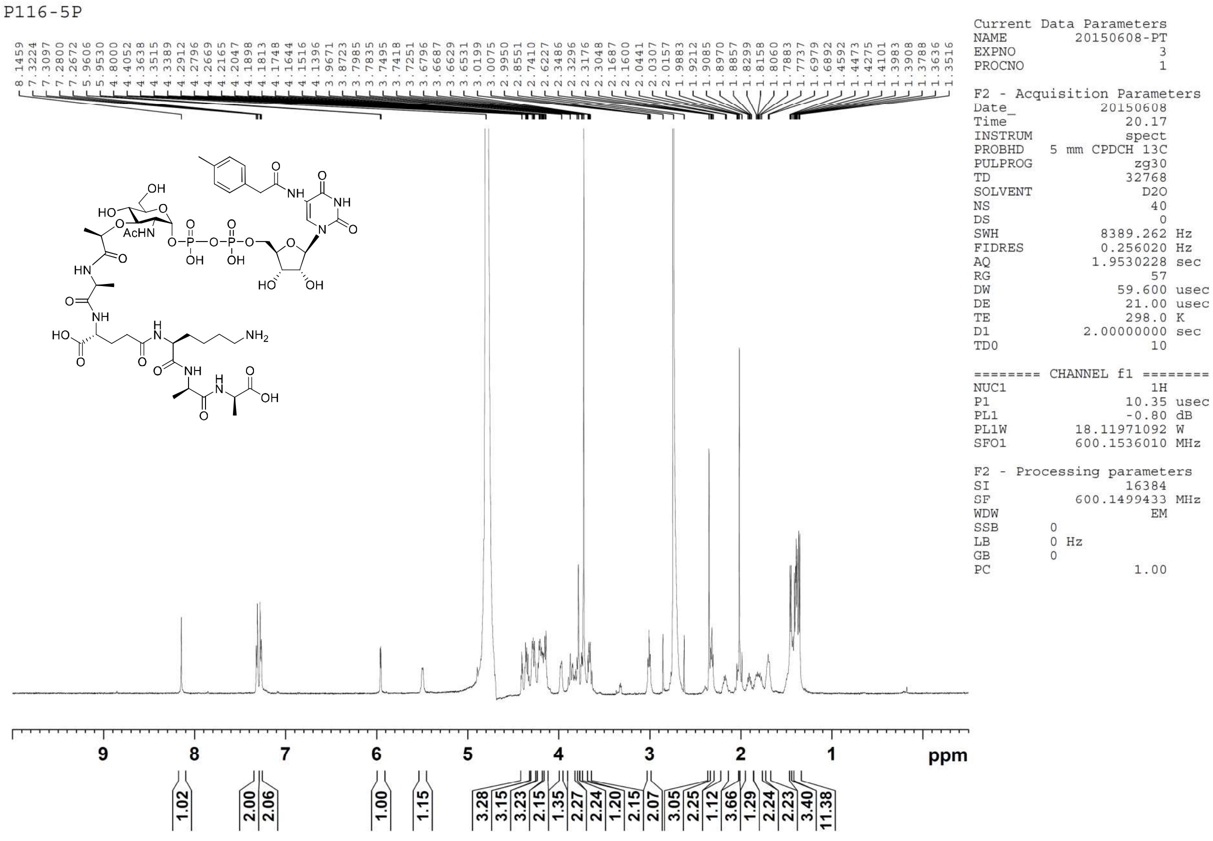

Supplementary figure 30. ^{1}H-NMR Spectra of compound **22** (600 MHz, D_2O)

References

1. C. Y. Liu *et al.*, Synthesis and Evaluation of a New Fluorescent Transglycosylase Substrate: Lipid II-Based Molecule Possessing a Dansyl-C20 Polyprenyl Moiety. *Org Lett* **12**, 1608-1611 (2010).
2. K. T. Chen *et al.*, Rapid preparation of mycobacterium N-glycolyl Lipid I and Lipid II derivatives: a biocatalytic approach. *Eur J Chem* **19**, 834-838 (2013).
3. A. J. Egan *et al.*, Activities and regulation of peptidoglycan synthases. *Philos Trans R Soc Lond B Biol Sci* **370**, 20150031 (2015)
4. Chang, Y. F. *et al.* Solid-phase organic synthesis of polyisoprenoid alcohols with traceless sulfone linker. *J Org Chem* **73**, 7197-7203 (2008).
5. P. E. Brandish *et al.*, Slow binding inhibition of phospho-N-acetylmuramyl-pentapeptide-translocase (Escherichia coli) by mureidomycin A. *J Biol Chem* **271**, 7609-7614 (1996).
6. J. L. Banks *et al.*, Integrated Modeling Program, Applied Chemical Theory (IMPACT). *J Comput Chem* **26**, 1752-1780 (2005).
7. B. C. Chung *et al.*, Crystal structure of MraY, an essential membrane enzyme for bacterial cell wall synthesis. *Science* **341**, 1012-1016 (2013).
